# Multifunction RF Systems for Naval Platforms

**DOI:** 10.3390/s18072076

**Published:** 2018-06-28

**Authors:** Peter W. Moo, David J. DiFilippo

**Affiliations:** 1 Defence Research and Development Canada–Ottawa Research Centre, Ottawa, ON K1A 0Z4, Canada; 2 Dannaven Inc., Ottawa, ON K1A 0Z4 Canada; djjdifilippo@gmail.com

**Keywords:** naval systems, multifunction RF systems, active electronically steered arrays, radar, electronic support, electronic attack, communications

## Abstract

The evolving role of modern navies has required increasingly higher levels of capability in the Radio Frequency (RF) shipboard systems that provide radar, communications, Electronic Attack (EA) and Electronic Support (ES) functions. The result has been a proliferation of topside antennas and associated hardware on naval vessels. The notion of MultiFunction RF (MFRF) systems has drawn considerable interest as an approach to reversing this trend. In a MFRF system, RF functions are consolidated within a shared set of electronics and antenna apertures that utilize Active Electronically Scanned Array (AESA) technology. This paper highlights a number of issues to be considered in the design and implementation of a naval MFRF system. Specifically, the key requirements of the RF functions of interest are first reviewed, and MFRF system design trade-offs resulting from costs and/or performance limitations in existing hardware technology are then discussed. It is found that limitations in hardware technology constrain the implementation of practical MFRF systems. MFRF system prototype development programs that have been conducted in other countries are described. MFRF resource allocation management is identified as an important future research topic.

## 1. Introduction

The evolving role of modern navies has required increasingly higher levels of capability in the Radio Frequency (RF) shipboard systems that provide radar, communications and Electronic Warfare (EW) functions, including in the latter case both Electronic Attack (EA) and Electronic Support (ES). The result has been a proliferation of topside antennas on naval vessels. It has been estimated that the number of topside antennas has roughly doubled on ships launched in the 1990s relative to those launched in the 1980s, with the antenna count on a typical 1990s-era destroyer for example being on the order of 80 [1]. This has led to a number of problems, including increased mutual electromagnetic interference, larger ship Radar Cross Section (RCS), and higher life-cycle costs associated with the operation of multiple unique RF systems.

Since the late 1990s, the idea of MultiFunction RF (MFRF) systems has drawn considerable interest as an approach to addressing this issue. In a MFRF system, several RF functions are consolidated within a shared set of electronics and antenna apertures. Active Electronically Scanned Array (AESA) technology is a key enabler for these systems. A modern AESA employs a separate transmit (Tx) and/or receive (Rx) channel for each of its radiating elements, with a high-power amplifier (HPA) and low-noise amplifier (LNA) in each of the transmit and receive channels respectively. Often, there is also some type of beamforming element in each channel, such as a phase shifter or true time-delay (TTD) circuit. The HPAs, LNAs and beamforming elements are typically packaged into Monolithic Microwave Integrated Circuit (MMIC) modules that are incorporated in the array structure to be as close as possible to the radiating elements, thereby minimizing system losses. In general, the AESA architecture allows dynamic reconfiguration of the antenna aperture, including partitioning of the array elements into subarrays, to form multiple simultaneous transmit and/or receive beams in independent directions with different beam patterns and waveforms. This provides the level of flexibility that is required to support multiple RF functions with the same antenna aperture. 

The use of shared hardware in a MFRF system facilitates intelligent control of the RF functions with common resource allocation management software. In general terms, an intelligent Resource Allocation Manager (RAM) in a MFRF system performs the critical task of adaptively allocating system assets to the RF functions based on the dynamically changing priorities and resource requirements of these functions within a given mission scenario and sensed RF environment. System assets under RAM control broadly comprise waveform generators, AESAs, receivers, communication modems and signal/data processing resources. The waveform generators, receivers and modems are largely digital and software controlled, which accommodates rapid reconfiguration of these assets to provide the waveform and receiver characteristics required by the supported RF functions.

**Figure 1:**
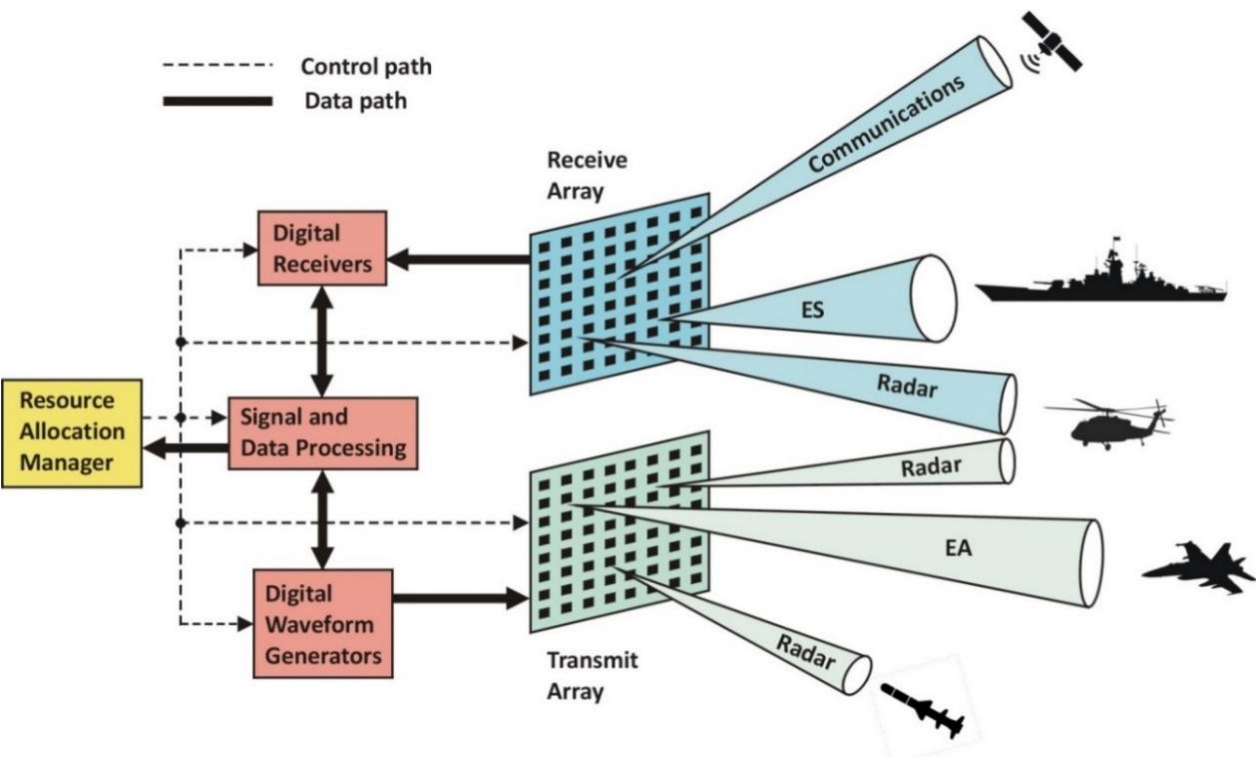
Conceptual diagram for MFRF system.

A high-level conceptual diagram of a MFRF system is shown in Figure 1. The general configuration depicted has separate receive and transmit AESAs, the advantages of which are discussed in Section 3. However, for certain MFRF implementations, a single aperture that combines both receive and transmit functions may be more desirable. The figure illustrates the notion that different sections of the apertures can be used to form simultaneous independent beams allocated to different RF functions. In this instance, a transmit beam is being used by the radar to illuminate an incoming anti-ship missile for the purpose of supporting the ship’s fire control system, while a second radar transmit beam is tracking a helicopter within its search volume. The EA function is utilizing a third transmit beam to jam the fire control radar of an approaching hostile fighter aircraft. With the receive array, a receive beam is formed in the direction of a satellite to establish a communication link. A second receive beam in the direction of a ship target is being used by the ES function. The radar is utilizing another receive beam to capture signal returns from the helicopter target that it is simultaneously illuminating. Note that the beamforming task is not explicitly broken out as a separate block in this figure because it is generally performed by a combination of the assets shown, depending upon the particular system implementation. Also, communications modems are not separately depicted, as their modulation and demodulation functions can conceptually be included in the waveform generator and receiver blocks.

MFRF systems can potentially provide the following benefits:

- Reduction of the ship RCS: By reducing the number of topside antennas, the aggregate contribution of the antenna apertures to the ship’s RCS is mitigated.
- Performance optimization of RF functions: In general, the overall performance of the suite of RF functions controlled by a central RAM is improved as a result of more tightly integrated scheduling of RF tasks. Of particular note, coordination of frequency usage between RF functions as part of waveform generation control by the RAM results in lower risk of mutual electromagnetic interference, as compared to the situation with separate RF subsystems where frequency management through less centralized control is generally suboptimal.
- Lower integration and life-cycle costs: The decrease in both the number of topside antennas and the amount of associated hardware can lead to less hardware integration effort and cost at the installation stage. Furthermore, the use of common hardware for the RF functions in a MFRF system can substantially reduce life-cycle costs as a result of requiring less unique spare parts, less maintenance training, and fewer personnel to operate and maintain equipment, relative to the situation with multiple single-purpose RF subsystems.

While MFRF systems may yield important benefits, it is also worthwhile to note a potential risk: the consolidation of RF functions within a fewer number of antenna apertures may increase vulnerability to a single point of failure. For example, if the topside antenna of a MFRF system is destroyed in battle, overall ship RF functionality may be more severely degraded than would be the case if the antenna supported only a single RF function. This risk would be considered in the cost/benefit analysis conducted to inform a decision on a MFRF system deployment.

This paper highlights a number of factors and challenges to be considered in the design and implementation of a naval MFRF system, and identifies MFRF resource management as a key topic for future research in this area. The next section reviews the requirements for naval radar, EA, ES and communications functions that have specific impact on MFRF system design. In Section 3, MFRF system design considerations and trade-offs are summarized. Specific MFRF system prototype development programs that have been previously conducted are described in Section 4. Section 5 provides a description of MFRF resource management, and conclusions are contained in Section 6. It is assumed throughout the paper that the reader is somewhat familiar with the underlying AESA theory and terminology. If not, one of a number of references can be consulted, such as [2,3,4,5].

## 2. Requirements for Naval RF Functions

The particular requirements for naval RF functions that most impact MFRF system design relate to transmit and/or receive specifications, since these are the requirements that can pose the greatest challenge to sharing a common set of electronics and antenna apertures between multiple RF functions. Table 1 provides a comparison of key transmit/receive requirements that are representative of naval radar, ES, EA and communications functions, with further explanation following the table.

A description of the terminology in Table 1 is provided below.

- *Frequencies of operation*: This refers to the range of frequencies over which the RF function is required to operate. For the radar function entries in the table, IEEE frequency band designations are used to characterize the frequencies of operation, as is a common practice in the radar field. These designations are defined in Annex A for convenience.
- *Signal bandwidth*: Signal bandwidth refers to the maximum instantaneous bandwidth of the signals that must be accommodated by the RF function. This requirement impacts design of the MFRF system receiver channels, in that the receiver analog bandwidth, as well as the sampling rate of the *a*nalog-to-*d*igital *c*onverter (ADC) needed to digitize the signal, must be at least equal to the instantaneous signal bandwidth. It is notable that one disadvantage to increasing the receiver bandwidth is that it leads to a larger system noise bandwidth, which may make noise-limited detection of signals more challenging. Another system design area that may be affected by this requirement is the array beam steering. If the instantaneous signal bandwidth is large enough, TTD beam steering may be necessary, as opposed to a simpler implementation with phase shifters.
- *Dynamic range*: This refers to the instantaneous dynamic range of the receiver, which is a metric that reflects the ability of a receiver to accommodate a range of input power levels from the antenna. It is calculated as the ratio of the strongest to weakest input power levels that can be properly measured by the receiver.
- *EIRP*: The Effective Isotropically Radiated Power (EIRP) is a standard measure of transmitted power that is calculated as $EIRP=PG_{t}$, where *P* is the peak transmitter power and $G_{t}$ is the antenna boresight gain upon transmit. For an AESA antenna, the peak transmitter power is determined by the sum of the peak power outputs from all of the HPAs utilized. As the HPA is often the most costly component in an AESA design, the EIRP requirement represents one of the most significant AESA cost drivers.
- *One*-*way beamwidth*: The one-way beamwidth requirement listed in Table 1 refers to the width of the beam formed upon transmit or receive, as measured between the −3 dB points of the beam mainlobe pattern. For naval radar and communications functions which employ both Tx and Rx beams, the transmit and receive beamwidths may be different if differently sized subarrays on the antenna aperture are used to form the transmit and receive beams, or separate Tx and Rx arrays of different sizes are employed. However, they are often the same in practice, and are assumed to be so for the purposes of this table. Narrower beamwidths allow more accurate localization and tracking of detected targets for RF functions such as radar and ES, but slow down the scanning process in a given search volume.
- *Duty cycle*: The duty cycle value in Table 1 is a characteristic of transmitted waveforms for those RF functions that involve signal transmission. It is calculated as τ/T where τ is the time duration of the transmitted waveform and *T* is the waveform repetition interval.
- *Signal Polarization*: The signal polarization that must be accommodated upon transmit and/or receive has a significant impact on the design of the required radiating elements in the array. For signals with a single linear polarization, a relatively simple radiating element design can be used, whereas for any other signal polarization, a more complex dual orthogonally-polarized element design is needed.

Note that in addition to the requirements listed in Table 1, there is a common requirement for all naval RF functions to provide full or almost full hemispherical coverage above the ship deck. Specific discussion of the requirements as they apply to the different RF functions is provided in the subsections below.

### 2.1. Radar Function Requirements

The naval radar function can be broadly divided into three main subfunctions: *v*olume *s*earch (VS), *h*orizon *s*earch (HS), and *t*erminal *i*llumination (TI). VS involves scanning the upper hemisphere above the ship deck to detect and track air targets primarily, although the coverage volume extends down to the horizon to also allow detection of larger surface vessels. Because air targets can be relatively fast, the VS subfunction is designed to provide a long-range detection capability so that incoming air threats are registered as soon as possible. HS more specifically focuses on detection and tracking of targets at low elevation angles out to the horizon, including both smaller surface targets and low-flying air targets. The design of this subfunction is driven primarily by the requirement to detect the supersonic sea-skimming *a*nti-*s*hip *m*issile (ASM) threat with sufficient warning time to deploy countermeasures. Compared to VS, the search ranges and volumes in HS mode are relatively small, but significant challenges exist in dealing with very small ASM RCS values, low-angle sea clutter, multipath interference nulls, and anomalous propagation conditions such as surface ducting. TI is a radar subfunction that supports onboard fire control systems when semi-active missiles are deployed. In this event, the radar must illuminate the target during the missile terminal guidance phase, so that the missile seeker can home in on the reflected signal.

As indicated in Table 1, VS typically operates at a frequency within L-band or S-band. These are the bands of choice for long-range detection since signals at these frequencies suffer less attenuation from precipitation than would be the case at higher frequencies. Conversely, HS most often employs higher S-band or X-band frequencies for primarily the following two reasons:

- At lower frequencies, there is a wide null in the radar propagation factor that forms in the elevation plane for small target elevation angles, due to multipath interference between the direct target signal return and the specular reflection of the signal return from the ocean surface. This would make detection in HS mode of certain targets like small surface vessels or sea-skimming ASMs particularly difficult [6]. Multipath nulls are present for this scenario at higher frequencies as well, but are narrower in elevation angle, and therefore have less of an adverse effect.
- The use of higher frequencies allows beamwidth and antenna gain requirements to be met with a smaller and lighter antenna, since beamwidth varies inversely as frequency, and antenna gain varies as the square of frequency. An important benefit of a smaller antenna is that it can be installed at a greater height above the ship’s deck to extend the distance to the horizon as much as possible, which is desirable in HS mode.

Finally, the TI function operates at an X-band frequency for compatibility with the seekers typically employed in semi-active missiles. Traditionally, multiple radars operating in different frequency bands have been installed on a vessel to collectively provide VS, HS and TI functionality. For example, an S-band radar would be employed for VS and HS, while a separate X-band radar would be used for TI. Alternatively, an X-band radar would provide both HS and TI, and another radar operating at L-band or S-band would be utilized for VS. 

Since radar returns are simply replicas of the transmitted waveforms, the instantaneous bandwidth requirements listed for the VS and HS subfunctions reflect the instantaneous bandwidths of their transmitted waveforms. The waveform bandwidths are selected to achieve a desired range resolution, where the highest obtainable range resolution ΔR is given by ΔR=c/2B, with c being the speed of light and B being the waveform bandwidth. The required instantaneous bandwidth for the HS function is generally somewhat larger than that for VS, because HS employs transmitted waveforms with higher range resolution. Better range resolution leads to a smaller effective radar resolution cell, since the resolution cell area $A_{c}$ for low radar grazing angles is given by $A_{c}\approx \Delta R\theta r$, where θ is the azimuth beamwidth and r is the radar range. A smaller radar resolution cell provides higher tracking accuracy, but more importantly for HS mode, improves the detection of slow-moving surface targets due to the fact that the amount of low-angle sea clutter returns that compete with target signal returns in the resolution cell containing the target is reduced. The larger instantaneous bandwidth required for HS mode implies a larger receiver bandwidth, which, as indicated previously, leads to a resulting increase in competing system noise power during the detection process. However, in the case of radar, the *s*ignal-to-*n*oise *r*atio (SNR) is not affected under these circumstances, as long as the waveform bandwidth is matched to the receiver bandwidth, and matched filtering, often referred to as pulse compression, is applied upon reception. Under these circumstances, the signal enjoys a proportionately higher pulse compression gain that allows the SNR to be maintained despite a larger value of system noise power. With regard to the TI function, the instantaneous bandwidth requirement is negligible, since the transmitted waveform is essentially monochromatic.

The receiver dynamic range requirements for the VS and HS subfunctions are determined by the ratio of the strongest expected clutter-plus-target return power to the weakest expected target return power. Dynamic range is not relevant for the TI subfunction since this radar mode has no receive component.

The naval radar function generally has the highest EIRP requirements relative to the other RF functions under consideration. For VS and HS subfunctions, the required EIRP is driven primarily by operational requirements that dictate maximum detection ranges for different types of targets. The operational requirements for VS typically involve longer detection ranges compared to HS, but on the other hand, some targets of interest in HS may have relatively smaller RCS values, especially for the ASM case. The required EIRP for operation of the VS subfunction at L-band is somewhat less than that for operation at S-band or X-band because of the better propagation characteristics of the lower frequencies, as noted earlier. It should be pointed out that the array receive gain $G_{r}$ also factors into the radar range equation to determine target detection capability of the VS and HS subfunctions, in that the SNR of the received radar signal is proportional to $EIRP\times G_{r}$. Consequently, increasing $G_{r}$ allows EIRP to be decreased proportionately without affecting detection ranges. For simplicity, the EIRP requirements listed in Table 1 for VS and HS are based on the assumption that the array receive gain is the same as the transmit gain, which would typically be the case for radar. The EIRP specification for the TI function is determined by the signal strength of the reflected target illumination required by the missile seeker for terminal guidance.

The requirements for one-way beamwidths listed in Table 1 are approximately the same for VS and HS radar subfunctions. The minimum achievable beamwidth is inversely proportional to array dimensions, so the beamwidth requirements directly impact the size of the AESA. For the VS function, required beamwidth values mainly derive from operational tracking accuracy requirements and required scan revisit times. These factors are considerations for the beamwidth specification of the HS function as well. However, as discussed earlier, the impact of radar resolution cell size on signal-to-clutter ratio for detection of surface targets additionally plays a role in arriving at the HS beamwidth requirement. In the case of the TI subfunction, there is no independent beamwidth requirement; the beamwidth used is just a consequence of the array size needed to provide the transmit gain that allows the EIRP requirement to be met.

The duty cycle requirements listed in Table 1 for the HS and VS subfunctions arise from a number of considerations, such as desired unambiguous range, size of range blind zone, and average waveform power, the latter of which is seen in the radar range equation to affect target detection. These duty cycle values are considerably less than the values listed for the TI subfunction and the other RF functions. In the case of the TI function, the illumination waveform is essentially narrowband continuous wave, which may have a transmission duty cycle of up to 100%, depending upon the capability of the semi-active missile seeker. Some semi-active missiles, such as the Evolved Sea Sparrow Missile [7], have seekers that support interrupted continuous wave illumination for terminal guidance, which allows the radar TI function to transmit with a duty cycle of less than 100%.

As indicated in Table 1, all radar functions typically use linear vertical polarization, primarily because there are distinct advantages to doing so for HS and no disadvantage to using vertical polarization for the other naval radar subfunctions. The advantages of using vertical polarization in HS mode are twofold. First, it has been observed that at radar grazing angles less than a few degrees (which is typically the case for the HS function) and higher sea states, sea clutter returns for vertical polarization are less than those for horizontal polarization [8]. Secondly, forward specular reflections off the ocean surface at low grazing angles are less for vertical polarization than for horizontal polarization due to the Brewster angle effect [9]. With lower specular reflections, the formation of multipath nulls in the radar propagation factor that adversely impact target detection at small target elevation angles is somewhat mitigated, although still remaining an issue.

### 2.2. ES Function Requirements

The ES function operates passively in a receive-only mode to monitor the RF environment around the ship. This function comprises automatic detection, analysis, identification, classification and angle-of-arrival measurement of received RF signals, especially radar signals. The analysis task from the above list specifically involves measurement of signal waveform parameters such as time of arrival, frequency, pulse repetition interval, pulse width and waveform modulation, and determination of threat radar parameters such as beamwidth and antenna scan revisit time. The ES function can often detect an approaching platform through its RF emissions before any other onboard sensor. Consequently, it serves to provide early threat alerts for emitters classified as hostile or unknown, and cues fire control systems if necessary with threat bearing information. Also, the EA function depends upon receiving information from the ES function to maximize its effectiveness, particularly threat bearing to enable it to point its beams accurately in the threat direction, and threat waveform parameters to allow optimization of the jamming technique. This support to the EA function is especially important when multiple threats are present simultaneously.

The frequencies of operation listed in Table 1 for the ES function refer to the frequency range over which the ES function is required to detect RF emissions. Emitters of interest include communications systems that typically operate below 5 GHz, surveillance and fire control radars which usually transmit in the 1–12 GHz range, and active missile seekers which mostly radiate at frequencies above 8 GHz.

As seen from Table 1, the typical instantaneous bandwidth required for the ES function is much higher than those listed for the radar subfunctions. This is largely a consequence of the potential need for an ES system to detect and properly characterize high bandwidth waveforms from emitters such as airborne synthetic aperture radars and low probability-of- intercept radars. It is worthwhile to note that unlike radar functions, there is no *a priori* knowledge of the received waveforms to allow matched filtering, and consequently, the increased system noise power due to large instantaneous receiver bandwidths results in lower sensitivity (i.e., lower SNR) that can adversely impact signal detection and analysis. The ES function can mitigate this impact for weak narrowband signals by digitally reprocessing the data captured within the wide instantaneous receiver bandwidth through a bank of narrowband filters before the detection process occurs.

The required receiver dynamic range for the ES function is based on the ratio of the strongest expected threat emitter power at the receiver input to an input level corresponding in strength to the receiver noise floor power.

There is no EIRP requirement for the ES function, as it is operates in a receive-only mode. The beamwidth requirement indicated in Table 1 applies to the beam that is formed by the AESA upon receive. The value for this requirement reflects the accuracy to which angle-of-arrival (AoA) of incoming emissions detected within a receive beam must be measured to allow useful determination of threat bearing. It should be noted however that a beam does not necessarily have to be formed by the AESA in order to determine AoA. If a strong RF signal arrives with a sufficiently high SNR, then the phases of the signal detected on a relatively small subset of array elements can be directly compared and processed with an interferometric algorithm to determine AoA with sufficient accuracy to meet the requirement. For such signals, this technique is relatively simple to implement, and essentially allows instantaneous detection and AoA measurement over the entire beamwidth of an array element, which typically represents a broad range of angles. However, for a weak signal, the antenna gain associated with the formation of a narrow receive beam may be needed to improve its SNR such that it can even be detected, with the AoA measurement in that case simply corresponding to the direction of the beam in which the detection is made.

Since duty cycle by definition is a feature of transmitted waveforms, there is no duty cycle requirement specified in Table 1 for the passive ES function.

In general, the ES function must be able to detect RF signals of all polarizations, due to the diversity of emitters that may represent potential threats. For example, many types of military communication systems use circular polarization. On the other hand, naval radars typically use vertical polarization for reasons discussed previously, while airborne radars usually employ horizontal polarization because sea clutter returns are significantly lower, compared to the vertical polarization case, at the larger radar grazing angles experienced by aircraft. The requirement to detect all polarization states dictates the use of dual orthogonally-polarized radiating elements in the AESA, which also gives the ES function the capability to measure the polarization characteristics of emitters as another identification attribute.

Due to its critical mission role, a unique requirement of the ES function is that it must always be fully operational, in the sense that it must continuously monitor the RF environment over the entire duration of the mission, without suffering any interruptions or performance degradation due to interference from the other onboard RF functions. In contrast, the other RF functions may be inactive at times, depending upon the scenario. Even the radar may be shut off during periods of covert operation.

### 2.3. EA Function Requirements

In general, a naval EA suite may utilize both onboard and off-board active devices, as well as off-board passive decoys, to provide platform self-protection by executing soft-kill countermeasures against threat RF systems. Since a MFRF system would be an onboard implementation, only the portion of the EA suite functionality represented by the onboard active device is considered for inclusion in such a system.

The EA function transmits a RF signal to jam a threat electronic system, which is usually a radar. The following generic categories of jamming techniques are typically employed:

- Noise jamming
- Multiple false targets
- Range gate pull-off (RGPO)
- Angle gate pull-off (AGPO)

Noise jamming of a threat radar is intended to prevent the hostile platform from detecting the ship or at least denying it range information until it gets much closer, making it more susceptible to a counterattack. Generation of multiple false targets within the coverage area of the threat radar makes it more difficult for the threat platform to identify or lock on to the ship, or can result in a threat missile being launched with less accurate targeting information. The goal of RGPO and AGPO is to lure the tracking gates of a threat radar off the ship’s signature in range and angle respectively. This leads to a break-lock situation in the case of a fire control radar, preventing the launch of a threat missile, while for the scenario of an active missile seeker being jammed, a successful AGPO causes the threat missile to miss the target.

In modern EA systems, these techniques are executed coherently, meaning that the jammer transmits a replica of the threat radar waveform based on digital samples of that waveform which have been captured and stored internally in a high-speed *d*igital *r*adio *f*requency *m*emory (DRFM) [10]. Consequently, the jamming waveform enjoys the same pulse compression gain in the threat radar as the original radar signal, thereby reducing the amount of jamming power needed to be effective. The jammer may also modify the replica before re-transmitting, such as adding a time delay to effectively impart a range offset to a false target. In the case of noise jamming, so-called “spot” noise can be generated with a bandwidth matched to that of the captured waveform in the DRFM, to ensure that the full jammer power is injected into the instantaneous bandwidth of the threat radar. 

The frequency range of operation for the EA function, as shown in Table 1, corresponds to the frequencies of a wide variety of threat systems that the EA function may be required to jam. The largest threats of concern are represented by active missile seekers, as well as surveillance and fire control radars that may be used to target the ship. It is seen that the frequencies of operation for the ES and EA functions are the same.

The instantaneous bandwidth requirement also mirrors that of the ES function, and refers in this case to the maximum bandwidth of the threat signal that it may need to replicate and transmit as part of a coherent jamming technique.

As a point of clarification, a conventional active EA system has its own receive channels and digitizers to capture and store threat system waveforms in DRFMs for implementation of coherent jamming, as mentioned above. However, as part of a MFRF system, the receive portion of the EA function would likely be provided by the ES function, since the frequencies of operation, instantaneous bandwidths and polarization requirements of the ES function are compatible with those of an EA receive subsystem. Consequently, the EA function is considered to operate in a transmit-only mode within a MFRF system, and therefore, has no dynamic range requirement.

The EIRP requirement specified for the EA function assumes the use of previously mentioned coherent jamming techniques, which maximize the effect of the available jamming power on threat radar systems. With coherent jamming, an EA technique will generally be successful to some extent if the jammer signal power arriving at the threat radar antenna is higher than that of the radar returns from the ship target. It is seen in Table 1 that the EIRP requirement for the EA function is significantly less than the corresponding radar subfunction values, where these values can be considered to be representative of threat radar systems as well. However, the EA function enjoys a relative signal power advantage that is proportional to $r^{2}$, where r is range to the threat radar, because of the fact that the jamming signal only suffers one-way geometric propagation losses proportional to $r^{2}$ along the path from the ship to the threat radar, as opposed to the two-way geometric propagation losses proportional to $r^{4}$ that are experienced by the threat radar returns from the ship. Consequently the jamming signal arriving at the threat radar antenna can be significantly stronger than the ship’s signature even though the EIRP value for the EA function may be less than that of the threat radar.

There is no independent beamwidth requirement for the EA function. The beamwidth is simply determined by the AESA size that provides the transmit gain needed to meet the EIRP requirement.

When the EA function is active, the duty cycle of its transmission may be up to 100%, as would be the case for example when AGPO is being attempted against a threat radar that is illuminating the ship with a CW waveform for the purposes of providing terminal guidance to a semi-active missile. The EA function cannot tolerate any interruptions while active.

The EA function is required to have the capability to transmit on any polarization. This is due to the fact that threat radar or communication systems may operate with linear, vertical or circular polarization, as mentioned in the ES function discussion of Section 2.2. The EA function may obtain the necessary information on the polarization state of the threat system from the list of threat signal attributes determined by the ES function. Alternatively, operation at the correct polarization is ensured if digital samples of the threat signal that have been captured on orthogonal polarizations by the ES function and stored in DRFMs are re-transmitted by the EA function.

### 2.4. Communication Function Requirements

In general, communication function requirements are fundamentally determined by the Shannon-Hartley Theorem, which states that:

$$C=B_{c}\log_{2}\left(1+\mathrm{SNR}\right),$$

where: C is the capacity of the communication channel, defined as the maximum achievable error-free data rate, in bits/second; $B_{c}$ is the channel bandwidth in Hz; and the signal-to-noise ratio SNR is defined as the ratio of the average received signal power to the average channel noise-plus-interference power, where it is assumed that the noise and interference can be characterized by Gaussian white noise. The received signal power is a function of the EIRP of the transmitting terminal, signal losses over the propagation path, and gain of the receive antenna. In lower frequency bands, where communication services have historically been located due to good signal propagation characteristics at these frequencies, data rates tend to be less because channel bandwidths are constrained by spectrum crowding. In higher bands, namely X-band and above, larger channel capacity is possible because greater spectrum availability allows wider channel bandwidths to be used. However, if the benefit of a wider channel bandwidth is to be realized, the EIRP and receive antenna gain of a shipboard communication terminal must typically be larger than those used in lower bands, in order to overcome the higher rain attenuation suffered at these higher frequencies along the signal propagation path.

A naval vessel employs different types of communication services that span frequencies up to K_a_ band. However, some of these services are not attractive candidates for inclusion in an AESA-based MFRF system. For example, there are a number of terrestrial-based services with low data rates, including voice and tactical data links (such as Link 16 and Link 22), which are operated at UHF frequencies or lower. These services typically employ omnidirectional monopole or dipole antennas, which are relatively simple and inexpensive, but must be large in length to provide efficient radiation at the low frequencies involved. These necessary antenna characteristics preclude such low-frequency services from consideration for an AESA implementation.

There are also requirements for a naval vessel to utilize communication services involving military satellite systems operating in UHF, X and K_a_ bands, as well as commercial satellite systems in L, K_u_ and K_a_ bands. Some examples include the military Wideband Global SATCOM system operating at X and K_a_ bands, and the commercial Telstar 12 VANTAGE system at K_u_ band. Compatibility with future V-band satellite systems may also be a requirement, but the characteristics of ground terminals for such systems are not yet standardized. Consequently, V-band systems will not be discussed here. A common characteristic of all existing communication satellite systems is the use of geostationary orbits. This results in very slow changes of the ship-to-satellite pointing angle, since the only contributor to such changes is the ship translational motion. UHF and L-band *sat*ellite *com*munications (SATCOM) terminals for shipboard application commonly employ simple low-gain helical and/or conical antennas to provide a hemispherical beam pattern, which removes the need for any pointing stabilization. Examples of such systems are the Thales QHASS UHF SATCOM terminal [11], which is currently on several of the Royal Canadian Navy Iroquois-class and Halifax-class vessels, and the L-band JRC JUE-87 Inmarsat C terminal [12]. These antenna packages are also compact, being less than 0.5 m in both diameter and height. Such low-gain antennas lead to EIRP values in the order of only 15 dBW, which are nonetheless sufficient because both communication data rates and rain attenuation of the RF signal are very low at these frequencies. Given the simplicity and compactness of these SATCOM antennas, their contributions to ship system life-cycle costs and ship RCS are already very small compared to those of other RF functions. Consequently, there is no obvious motivation to add UHF and L-band SATCOM services to an AESA-based MFRF system.

Current shipboard terminals for SATCOM services at the higher X, K_u_ and K_a_ bands typically use parabolic reflector antennas, commonly referred to as “dish” antennas. An example of this is the Thales SURFSAT-L naval SATCOM terminal [13], which is a dual-band system that can accommodate any two of the three specified bands. Consequently, a minimum of two such terminals is needed to cover all three bands. The antenna is isolated from ship roll, pitch and heading changes through a gimbal mounting with 3-axis or 4-axis inertial stabilization. It is noted that the diameter of the parabolic reflector needs to be in the order of 1–2 m to achieve the required gain at the frequencies of interest. Consequently, the RCS presented by such an antenna to a threat radar may be significant. For example, the maximum RCS of a parabolic reflector with a diameter of 1.5 m would be approximately 15 dBm^2^, as seen by an L-band threat radar [14]. These terminal antennas also occupy large physical footprints–the Thales SURFSAT-L antenna assembly above deck, including the radome, gimbal mounts and a parabolic reflector with a diameter of 2.1 m, has an overall diameter of about 2.7 m, a height of 3 m, and a weight of 380 kg. These considerations motivate incorporation of the X-band, K_u_-band and K_a_-band SATCOM services in a MFRF system.

A communication service referred to as Tactical Common Data Link (TCDL) is another common requirement for naval vessels. TCDL is a secure K_u_-band data link developed by the US military to send data and images from airborne platforms to surface platforms. It utilizes the Common Data Link (CDL) waveforms and protocol that have been mandated by the US Department of Defence as the wideband communications standard for transferring intelligence, surveillance and reconnaissance (ISR) sensor data between both manned and unmanned platforms. A typical example of a currently available shipboard TCDL antenna is the CPI SST-100 [15]. The entire above-deck antenna assembly, including a radome, stabilization gimbal mounts and a parabolic reflector antenna with a diameter of 0.8 m, has an overall diameter of 1 m, a height of 1.1 m and a weight of 200 kg. An antenna such as this would be needed for each independent TCDL link. Studies within the US Navy have indicated that at least four independent TCDL links are currently required, with future projections of up to 24 independent links [16]. Given the physical size of these parabolic reflector assemblies, the amount of available deck/superstructure space on a ship significantly limits the number of TCDL links that can be currently supported. Migration of the TCDL function to an MFRF system would alleviate this constraint, by virtue of the flexibility afforded by AESA use.

To summarize then, the communication functions that are considered for candidates in a MFRF system comprise the SATCOM services in the X, K_u_ and K_a_ bands, and the TCDL service. Referring to the frequencies of operation for these functions in Table 1, it is noted that the Rx and Tx operations are conducted over separate non-overlapping frequency sub-bands. This allows simultaneous reception and transmission, referred to as full duplex communication, on one antenna without causing self-interference. There is some further discussion provided on this issue in Section 3.

The instantaneous bandwidth requirements listed in Table 1 correspond to the channel bandwidths that are specified for the different communication services.

The dynamic range requirements for the communication services are largely driven by the variation in signal input levels corresponding to advanced high-order Quadrature Amplitude Modulation (QAM) schemes that support increasingly higher bit rates.

The shipboard EIRP requirements in Table 1 for the communication services reflect values that are necessary to compensate for rain attenuation of the signal at these frequencies, and for the one-way geometric propagation loss over the signal path, such that the SNR at the off-board receiver is high enough to support the desired channel capacity. The EIRP requirement for the TDCL function is noticeably lower than that for the SATCOM services primarily because TDCL is specified to operate over ranges of only up to 200 km, whereas the one-way signal propagation distances involved in SATCOM are approximately 36,000 km for geostationary satellites. 

The one-way beamwidth values that appear in Table 1 are not independent beamwidth requirements. Rather, as the antenna gain can be computed from the beamwidth, the beamwidth values serve to indicate in this case the associated antenna gains required to ensure that the SNR value of the signal received at the shipboard receiver is consistent with the desired channel capacity.

The transmission duty cycle of a communication function may be up to 100% when the function is activated. The polarization requirements listed in Table 1 are somewhat different depending upon the service. A common characteristic of the SATCOM services is that Rx and Tx polarizations are orthogonal, where orthogonality is achieved for linear polarizations by using horizontal and vertical polarizations, and for circular polarization by using right-hand circular polarization and left-hand circular polarization. The use of polarization orthogonality provides added isolation between Rx and Tx channels beyond that afforded only by duplex operation. More importantly however, this capability allows a SATCOM satellite to use orthogonal polarizations within the same frequency channel. This frequency re-use doubles the satellite’s communication capacity within a given bandwidth constraint, which is especially important for the crowded Ku spectrum. Orthogonal polarizations are not required for TCDL. Circular polarization is used for X-band SATCOM because at X-band frequencies and lower, Faraday rotation of linear polarizations in the ionosphere [17] causes unacceptable leakage between orthogonal polarization states, which adversely affects frequency re-use. In contrast, horizontal/vertical polarizations are employed for K_u_-band SATCOM because Faraday rotation is not an issue for linear polarization at K_u_ frequencies and above, and although rain causes leakage between all orthogonal polarization states at these frequencies due to the interaction of the SATCOM signal with large flattened raindrops along the propagation path, the leakage is less pronounced for orthogonal linear polarizations [18]. Note that the use of linear polarization requires precise stabilization of the shipboard antenna in the presence of ship motion to prevent misalignments between the shipboard antenna and satellite antenna that can contribute to leakage between polarization states. Notwithstanding the depolarization effects of rain described above, circular polarization is used for K_a_-band SATCOM to address a greater concern. Signals at K_a_ frequencies are generally more severely attenuated by rain than K_u_ and lower frequencies, and in particular, there is a more significant difference in attenuation between orthogonal linear polarizations at K_a_-band for large raindrops, due to the fact that they have dimensions comparable to K_a_-band wavelengths [18]. Specifically, the polarization along the axis of the widest dimension of the flattened raindrop is more attenuated than the orthogonal polarization. Because the angle of fall of the raindrops along the propagation path of the SATCOM signal is unknown, extreme signal fading due to an unfortunate alignment of linear signal polarization with the raindrop shape may occur. This situation is mitigated with circular polarization. Finally, TCDL employs circular polarization due to the difficulty of ensuring that shipboard and airborne antennas remain sufficiently aligned with each other during the communication link to allow linear polarization to be used. Shipboard antennas are fully stabilized in all three degrees of freedom, but airborne TCDL antennas are often stabilized in only azimuth and elevation to reduce the physical size and weight of the antenna assembly, particularly for a platform like a helicopter or unmanned aerial vehicle (UAV) where space is scarce. An example of this is the Honeywell AC-27 airborne TCDL antenna [19]. Consequently, certain types of motion experienced by the airborne platform with this type of antenna could result in excessive misalignment between the airborne and shipboard antenna orientations, leading to signal fade if linear polarization is employed. Use of circular polarization avoids this problem.

## 3. MFRF System Design Considerations

### 3.1. Ideal MFRF System Architecture

To provide context for the discussion in this section, it is useful to consider the key features of an ideal MFRF system architecture that would allow all AESA elements and Tx/Rx channel hardware to be utilized by any of the RF functions of interest. Such a configuration would maximize the potential benefits discussed in Section 1.

**Figure 2:**
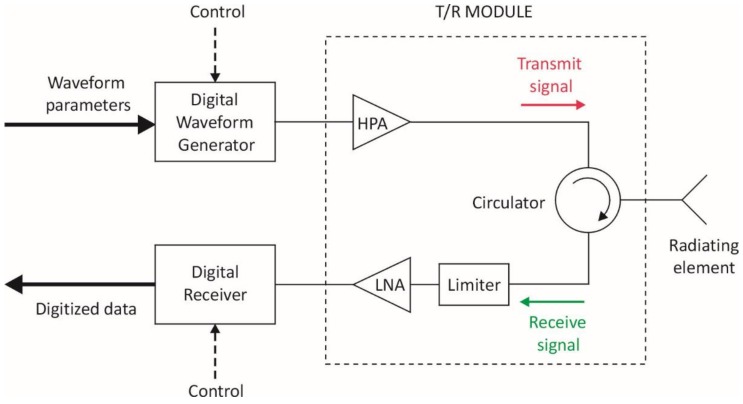
Block diagram of a single Tx and Rx channel in an idealized MFRF system.

The principal hardware components of a Tx and Rx channel in an ideal MFRF system architecture are depicted in the simplified block diagram of Figure 2 for a single AESA element. If the radiating elements are dual-polarized, each element polarization has a similar Rx and Tx channel associated with it.

The above figure conveys the notion that each radiating element is shared by a Tx channel and a Rx channel, where generally each of the two channels can be used by a different RF function at the same time. The circulator is the key element that enables this configuration. A circulator is a nonreciprocal three-port device that utilizes the properties of certain magnetic materials like ferrite to allow an RF signal to pass between ports in ideally only one direction, while preventing the signal from proceeding in the reverse direction around the circulator. So referring to Figure 2, the transmit signal entering the circulator is routed to the radiating element, and blocked in the reverse direction so that it does not emerge from the circulator into the Rx channel. Thus, the circulator serves to isolate the sensitive LNA in the Rx channel from the high power output of the HPA during transmission. This is important because while the limiter in the Rx channel is designed to attenuate excessively large RF power inputs that could cause LNA damage, signals at the input of the LNA may still be large enough to saturate the LNA, resulting in nonlinear amplification and resulting distortion of any input signals. Signal distortion could lead to adverse effects such as increased bit error rates for SATCOM signals or degradation of ES function capability to characterize modulation attributes of a threat radar signal. Use of the circulator also ensures that a RF signal entering the circulator from the radiating element is sent to the receive channel only, thereby providing isolation between the radiating element and HPA under these circumstances. Otherwise, RF energy may potentially be fed back into the HPA due to external signals picked up by the radiating element, or reflections from the radiating element as a result of element impedance mismatches. If such signals are large enough, the HPA may be damaged and/or suffer performance degradation [20].

Typically, the circulator, limiter, HPA and LNA are packaged together as part of a *t*ransmit/*r*eceive (T/R) module that is located close to the radiating element to mitigate system losses. In an ideal system, these T/R module components, along with the associated radiating elements, would have a sufficiently large bandwidth to accommodate the operating frequencies of all RF functions which may use the Tx and Rx channels. Based on the information in Table 1, this implies that operating frequencies of the radiating elements and T/R module components must extend from 0.5 GHz to 40 GHz for a naval MFRF system.

Each radiating element of an array used in an ideal MFRF system is also dual orthogonally polarized so as to support all polarization states. This is necessary to meet all of the polarization requirements listed in Table 1, particularly those for the EA and ES functions. To prevent grating lobes in the array gain pattern for typical maximum array scan angles of ±60°, the radiating elements are spaced at a distance of 0.54λ*_g_*, where λ*_g_* is the wavelength value at the highest frequency of operation. From Table 1, the highest frequency is 40 GHz, implying an element spacing of 0.4 cm.

In an ideal architecture, each Tx channel has its own digital waveform generator (DWG), which can also access a DRFM to support coherent jamming for the EA function. A DWG performs the following steps: receive arbitrary waveform parameters from the signal/data processor of the MFRF system; digitally generate samples of the desired waveform with a *d*irect *d*igital *s*ynthesizer (DDS), or extract waveform samples from a DRFM; use a *d*igital-to-*a*nalog *c*onverter (DAC) to convert the samples to the analog domain; and finally, translate the waveform to the required frequency band with analog modulation circuitry. The presence of a DWG in each Tx channel provides the ultimate flexibility when using the array for transmission, in the sense that from one point in time to the next, the array can be instantly repartitioned through software control into Tx subarrays of arbitrary size, with each subarray forming an independent Tx beam for a RF function. This high level of array reconfiguration capability facilitates optimal MFRF system performance under normal circumstances, and graceful performance degradation if parts of the array suffer failure. A second key advantage of including a DWG in each Tx channel is that TTD beamforming can be readily achieved by digitally introducing a time delay between identical waveforms generated for adjacent radiating elements in a subarray. This approach ensures that Tx beams employing wideband waveforms can enjoy maximum array gain when steered off boresight, without the need for analog TTD circuits elsewhere in the Tx channels. TTD beamforming is necessary primarily for the EA function, since the instantaneous bandwidth of waveforms transmitted by this function may be as high as 1 GHz. Finally, a DWG in each channel also provides a straightforward means to weight the waveform amplitude across the subarray elements for sidelobe control or adaptive placement of nulls in the Tx beam.

The digital receiver in each Rx channel contains an ADC that digitizes the signal received from the radiating element. This element-level digitization permits receive beams to be formed entirely in the digital domain, that is, without the need for RF phase shifters or TTD circuits elsewhere in the Rx chain. This scheme has three key benefits. First, many simultaneous receive beams can be formed in different directions using the same set of array elements, thereby providing instantaneous coverage of a large volume. The maximum number of these independent simultaneous beams is limited only by available signal processing power. This is a particularly useful capability for the ES and radar functions which must detect and localize threats as quickly as possible within a large surveillance area. Secondly, TTD formation of receive beams can be easily realized in software by introducing time shifts between digitized signals from adjacent array elements before coherent summation across the elements. TTD beamforming on receive is important for the ES function, which must be capable of detecting and characterizing signals of instantaneous bandwidths up to 1 GHz. For such signals, conventional beamforming with phase shifters would result in reduced array gain at scanning angles off boresight, compared to TTD beamforming which provides the highest achievable array gain at all scan angles for signals of any bandwidth. Consequently, TTD beamforming optimizes detection for a MFRF system. Finally, element-level digitization, similar to the benefit provided by a DWG in each Tx channel, allows the maximum flexibility in allocation of array resources to receive operations associated with the different RF functions.

In this ideal case, the element-level digitization is done directly at RF. To accommodate all RF functions of interest, this implies a required ADC sampling rate capability of 40 *g*iga*s*amples *p*er *s*econd (GSPS), corresponding to the highest frequency of operation listed in Table 1. The ADC in each digital receiver must also have sufficient dynamic range to meet the most stringent dynamic range requirement indicated in Table 1. This corresponds to a dynamic range value of 90 dB for radar functions, which implies about 15 bits of digitization. Once the Rx signal is digitally captured in this way, all other traditional receiver functions, such as filtering and quadrature demodulation, can be done through digital signal processing. Furthermore, the same digitized set of signal data can be processed in parallel in different ways, to meet the needs of different RF functions. For example, a digitized signal data set can be digitally filtered at the frequencies utilized by the VS radar function (assuming that there was a corresponding VS radar transmission to generate radar return signals), and pulse compressed as the first step in the target detection process. Concurrently, for the ES function, banks of bandpass filters spanning the full ES monitoring range can be applied to the same digitized data, using different filter bandwidths to mitigate the effect of system noise on detection of both wideband and narrowband weak emitter signals. This scheme would essentially allow instantaneous searching of threat signals over the entire frequency range of interest, eliminating the need for a separate *i*nstantaneous *f*requency *m*easurement (IFM) receiver typically required in many current ES systems.

### 3.2. Practical Limitations and Trade-Offs

The ideal MFRF system architecture described above is not currently achievable in practice, due to costs or performance limitations in existing hardware technology that force design trade-offs and compromises to be made. The trade-offs broadly fall into three categories: (1) combined vs. separate Tx/Rx arrays [21]; (2) wideband vs multiband operation [22]; and (3) element-level vs. subarray-level digitization/waveform generation. It is expected that all three of these trade-offs would factor into a system design. These trade-offs and their implications, which are further explored in the subsequent sections, generally result in a larger number of AESAs and less flexibility in array utilization than would be the case for the ideal architecture.

Some comments on costs are also included in the following discussion. However, meaningful estimates and comparisons of system-level costs cannot be provided without detailed system designs, which are beyond the scope of this study.

There is no discussion in this section of potential constraints in system design and performance that may be imposed by signal/data processing resources. This type of technology continues to advance rapidly, driven by requirements in diverse fields such as artificial intelligence, cloud computing and gaming. For example, Graphics Processing Units (GPUs), which have been developed and used extensively for all three of these applications, are also well-suited for use in massively parallel architectures that exhibit the large data throughput, high computational performance, low latency and easy scalability required by AESA-based radar signal processing algorithms [23]. Given its continued fast pace of development, computing technology is consequently viewed as a much less significant limiting factor for MFRF system performance than the other hardware issues that will be addressed below.

#### 3.2.1. Combined vs. Separate Transmit/Receive Arrays

In practice, a wideband microstrip circulator that may be used in a T/R module does not completely suppress signals travelling in the reverse direction around the circulator. At best, currently available circulators can provide about 30 dB of such suppression between circulator ports, as exemplified by the JC2S8000T12K0G2 microstrip circulator (JQL Electronics, Rolling Meadows, IL, USA) [24] which operates over 8–12 GHz. This is sufficient for isolation of the HPA from signals returned by the array element, but not for isolation of the LNA from the HPA output. The issue is that for a typical wideband LNA, exemplified by the HMC1049LP5E LNA (Analog Devices, Norwood, MA, USA) for instance, the input power corresponding to the 1-dB gain compression point is about 1 mW [25]. The 1-dB compression point on the LNA gain curve indicates the point at which the LNA starts to become saturated. With a circulator providing 30 dB of isolation between Tx and Rx channels, the peak HPA output power must consequently be less than 1 W to ensure that leakage into the Rx channel does not drive the LNA into saturation if transmitting and receiving simultaneously. For some naval RF functions with a high EIRP requirement, such as radar, this is an onerous constraint. Normally, naval radars employ T/R modules with peak powers of at least 10 W to achieve the required EIRP with a reasonable number of modules. Consequently, usage of lower power T/R modules to accommodate simultaneous transmitting and receiving would imply the need for many more such modules to meet EIRP requirements, leading to increased cost, size and weight of the array.

A potentially more serious issue relates to power reflected back from the radiating element during transmission due to impedance mismatches between the antenna element and free space. This reflected power emerges unattenuated from the circulator into the Rx channel, and as such, represents another source of leakage from the Tx channel. In a traditional mechanically scanned antenna, where the residual impedance mismatch is only a function of frequency, this mismatch can be electronically tuned out near the antenna to mitigate the problem. However, with an AESA, mutual coupling between radiating elements in the array result in an element impedance mismatch that varies both with frequency and scan angles, making it much harder to control [2]. This leads to element reflection coefficients that can be as high as −6 dB when measured over a ±60° scan sector and across a wide range of frequencies [26]. Assuming the same LNA characteristics as in the previous paragraph, and a −6 dB element reflection coefficient, the peak HPA output power must be less than 4 mW to ensure that this reflected power does not saturate the LNA during simultaneous transmitting and receiving. Along the lines of the discussion in the previous paragraph, this would clearly impose a problematic limitation on AESA design with regard to those RF functions with high EIRP requirements.

If separate Tx and Rx arrays are used, Tx and Rx channels are largely electrically disconnected. In this case, the principal source of leakage becomes *e*lectro*m*agnetic (EM) coupling between the two arrays, which can be reduced to an acceptable level simply by increasing the physical separation between them. For example, with a separation of a few metres between edges of a Rx and Tx array in the same plane, isolation values of greater than 80 dB can be readily achieved over a wide range of frequencies and array scanning angles [27]. With this level of isolation, high-power HPAs can be used on the Tx array without affecting simultaneous reception on the separate Rx array.

It should be pointed out that a combined Tx/Rx array, meaning an array that uses T/R modules to enable sharing of each array element by a Rx and Tx channel, can be configured to emulate separate Tx and Rx arrays by utilizing two subarrays on the same aperture with a separation between them. In this scenario, only the Tx channels of the T/R modules for one subarray and only the Rx channels for the other one would be employed. Consequently, the EM coupling between the two subarrays would be the only contributor to leakage from the Tx to the Rx channels. EM simulation results at C-band have been reported for this type of configuration, involving two small subarrays separated by about 2 m on an array of wideband flared notch elements [28]. The simulations indicated acceptable isolation between the subarrays of at least 70 dB, modelled with the Tx subarray beam pointed at boresight and the Rx subarray beam scanning over ±60° in both azimuth and elevation. However, a potential problem with this approach is that given the fixed size of the array, the only way to increase subarray separation to achieve the isolation required for simultaneous transmission and reception is to reduce the size of the subarrays. This leads to lower EIRP (due to a smaller numbers of T/R modules in the subarray and lower subarray gain) and larger widths for the Tx and Rx subarray beams. As a result, the subarray EIRP and beamwidths may fail to meet the requirements of the RF functions for which the subarrays are to be used.

Another potential advantage of using separate Tx and Rx arrays is that it affords greater design flexibility than that which is possible with a combined Tx/Rx array. For example, antenna gain and beamwidth, which are functions of the AESA size, can in general be different for Rx and Tx operations. Separated arrays allow for the possibility of differently sized Rx and Tx arrays to be used to achieve this. Also, with separated Tx and Rx arrays, different technologies in principle can be more easily employed for fabrication of the Tx and Rx modules with which the respective arrays would be populated. For example, older gallium arsenide (GaAs) technology could be used for Rx modules since it is well suited for the fabrication of low-noise wideband LNAs. On the other hand, newer gallium nitride (GaN) technology, while currently more expensive than GaAs technology, is attractive for use in Tx modules, since it allows HPAs to be fabricated with five times more power output than GaAs HPAs within the same chip footprint [29].

The obvious disadvantage of employing separate Tx and Rx arrays in a MFRF system is that the number of required antenna arrays would be greater than that needed for the case where combined Tx/Rx arrays are used. The general rule of thumb is that AESAs provide useful coverage over scan angles of $\theta_{0}=$ ±60° relative to boresight. This results from the practical fact that impedance matching of AESA elements over large scan angles becomes increasingly difficult, and also from the theoretical observation that for any AESA antenna, the antenna gain varies as $\cos\theta_{0}$ and the beamwidth varies as ${\left(\cos\theta_{0}\right)}^{-1}$. At $\theta_{0}=\pm$60°, the resulting antenna gain drop of 3 dB and the beamwidth increase factor of 2 start to become significant. Consequently, if combined Tx/Rx arrays are used, at least three such arrays are required to provide hemispherical coverage, and often four are preferred to minimize performance degradation at the edge of the scan patterns. Now considering the case of separate Rx and Tx arrays, these numbers would be doubled, adding at minimum another three topside antennas to provide the required coverage volume. Thus, the use of combined Rx/Tx arrays mitigate to a larger extent the contribution of topside antennas to overall ship RCS, and may simplify antenna installation due to the fewer number of antenna arrays involved.

It is worthwhile to note that for stand-alone naval radar systems or communication systems, the problems with combined Tx/Rx arrays discussed in this section are not necessarily relevant. For naval radars, which typically use a pulsed waveform with a relatively low duty cycle, there is no requirement to receive while transmitting. Consequently, an electronic switch can be included in the Rx path between the LNA and circulator, and opened during radar pulse transmission to provide sufficient isolation for the LNA. In the case of fully duplexed communication systems, transmission and reception are conducted on different frequency bands, as indicated in Section 2. As a result, any residual HPA output power leaking from the Tx channel into the Rx channel through the circulator would be outside of the Rx band, and can consequently be attenuated by a bandpass filter inserted into the Rx channel between the LNA and circulator. Furthermore, radar and communication functions are assigned different frequency bands of operation, so that they would generally not interfere with each other in a MFRF system. It is only when the ES and EA functions are included in a MFRF system that isolation between Rx and Tx channels becomes critical, because the ES function must continuously monitor a large range of frequencies including those at which the other RF functions may be simultaneously transmitting, and the EA function may be required to transmit at frequencies over which the other RF functions are simultaneously receiving. This discussion suggests a possible MFRF system design compromise, in which radar and communication functions share a combined Tx/Rx array, while the EA and ES functions employ separate Tx and Rx arrays, respectively, to enable simultaneous transmission and reception.

#### 3.2.2. Wideband vs. Multiband Operation

In the ideal MFRF system architecture, all of the hardware components are sufficiently wideband to support the full range of operating frequencies for the RF functions of interest. This implies component bandwidths of 0.5–40 GHz, based on the RF function requirements of Table 1. However, such bandwidths are not available with current state-of-the-art technology.

**Figure 3:**
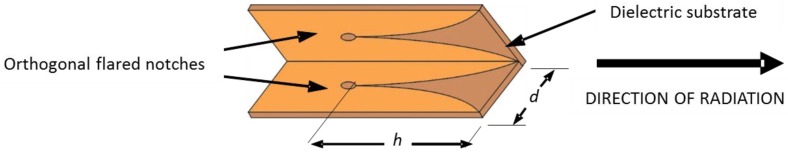
Dual polarized AESA element based on flared notches.

Referring to Figure 2, limitations begin with the radiating element itself. A number of different types of antenna elements have been designed for use in dual-polarized wideband AESAs, but the element design that provides the largest bandwidth along with relatively good cross-polarization and reflection coefficient properties remains the well-known flared notch, often referred to as a Vivaldi antenna [26,30]. Figure 3 depicts a single AESA element comprising two flared notches positioned orthogonally to provide dual linear polarization operation. The metal flared notches can simply be printed on dielectric substrates, which facilitates cost-effective fabrication and assembly of a large array of such elements, with small element separation if necessary. The bandwidth is largely determined by the element aspect ratio *h*/*d*, while the element separation on the array corresponds to the dimension *d*. The maximum instantaneous bandwidth achievable with a flared notch element occurs with an aspect ratio of *h*/*d* ≈ 5, which yields a bandwidth of about 10:1, or a decade of bandwidth. However, the overall required 0.5–40 GHz range of operating frequencies for the naval RF functions represents almost two decades of bandwidth. Consequently, the limitation on achievable AESA element bandwidth necessitates the use of multiple AESAs operating in different bands that collectively cover the entire operating band of interest.

**Figure 4:**
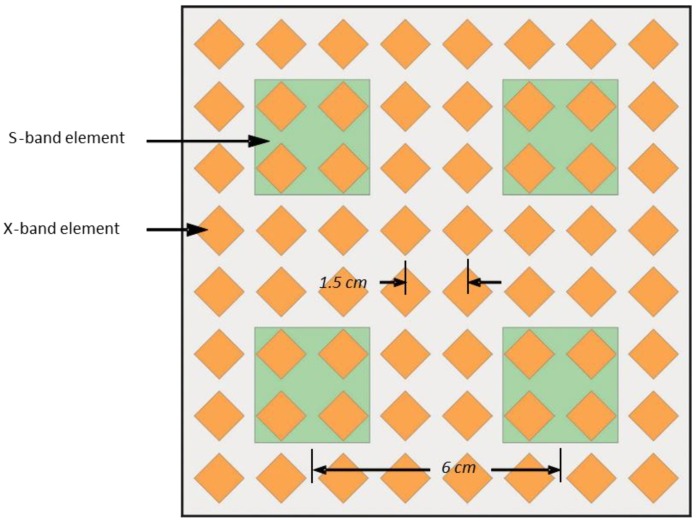
Geometry of dual-band array.

In considering various multiband MFRF system designs, one design approach is to populate every AESA with elements that are as wideband as possible, with the aim of accommodating all RF functions that operate within the large bandwidth of each array. An alternative scheme could include some AESAs that are designed to be utilized only by RF functions that have relatively small operating frequency ranges, albeit in different bands. This potentially allows narrowband elements to be used for those arrays. For example, a dual-band dual-polarization AESA design has been reported that supports operation at both S-band and X-band [31]. The geometry of the array is shown in Figure 4. The spacing of the S-band and X-band elements is chosen to minimize grating lobes in their respective bands. The radiating elements themselves consist of metal patches printed on different sides of four stacked dielectric substrates. The separation distance between the stacked substrates is adjusted to optimize the bandwidth of the elements. Each X-band element comprises two stacked diamond-shaped patches–one patch is active and the other is parasitic. There are two feed ports to the active patch on adjacent corners to realize dual orthogonal polarizations. Each S-band element consists of stacked patches that include two modified coupling feed patches, an active perforated patch and a parasitic perforated patch. The purpose of the perforations is to expose four X-band elements that underlie each S-band element, so that the presence of the S-band element does not affect the performance of the X-band elements. The measured bandwidths of the S-band and X-band elements are 0.6 GHz and 2.7 GHz respectively, corresponding to operating frequency ranges of 2.8–3.4 GHz and 9.0–11.7 GHz. The element reflection coefficient is less than −10 dB over these frequency ranges. This type of AESA would appear to be a good candidate for shared usage by the S-band and X-band radar subfunctions listed in Table 1. The array design provides the flexibility of allowing any part of the AESA to be accessed simultaneously by radar functions in both bands. Furthermore, the employment of relatively narrowband radiating elements in the AESA yields the attendant benefit of being able to readily source narrowband components with the required performance characteristics for the associated Rx and Tx channels.

**Figure 5:**
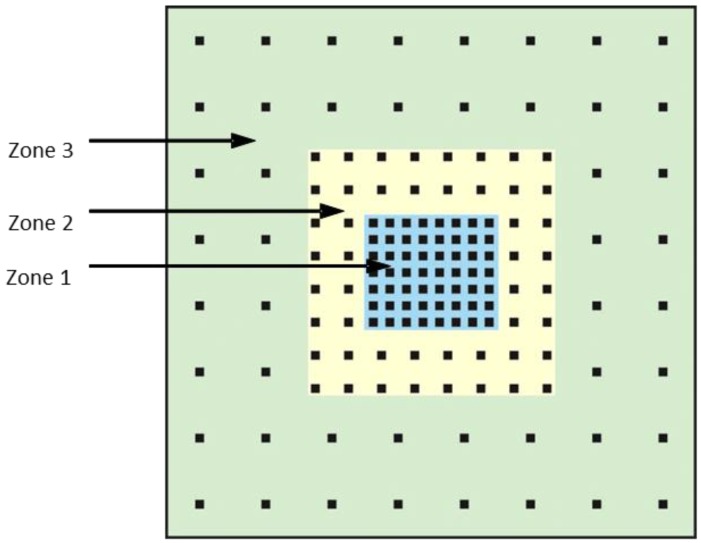
Wavelength-scaled array concept.

Another AESA-related issue is the separation between elements on the array. As indicated in Section 3.1, in order to prevent grating lobes within the full operating frequency range and scan angle sector of ±60°, this spacing should be 0.54λ_g_. However, assuming a wideband AESA with a 10:1 bandwidth, this means that for all but the highest frequency within the supported bandwidth, the number of elements populating the AESA would be more than required, by a factor between one and 10. This is a concern because the cost of an AESA implementation is largely proportional to the number of elements used, with the cost of the Rx and/or Tx channel electronics associated with each element being the main cost driver. To appreciate the scale of the problem, consider a MFRF system utilizing an AESA with a 10:1 instantaneous bandwidth covering 1–10 GHz. Referring to Table 1, if the radar volume search function is being conducted in L-band at 1 GHz, then the one-way beamwidth requirement of 2° for that RF function dictates that the array size is about 7.6 m per side. If the element spacing is then set to 0.54λ_g_ to avoid grating lobes at 10 GHz, then a total number of 220,000 radiating elements is required for the AESA. One way to reduce the overall element count is to further divide the frequency range of interest into multiple smaller bands, each band with its own AESA. This allows the AESAs covering lower frequency bands to utilize less elements as a result of larger allowed element spacing. However, this solution carries with it all the previously mentioned disadvantages of additional antenna apertures that need to be installed on the ship. An alternative multiband approach that maintains use of the single wideband array with the same overall size and bandwidth is based on the implementation of different element spacing in various zones on the AESA. This idea has been referred to as a wavelength-scaled array [32]. The concept is depicted in Figure 5 which indicates the element locations within the different zones on a wavelength-scaled AESA. In this example, the element spacing in Zone 3 is twice that of Zone 2, which, in turn, is twice the element spacing of Zone 1, where the Zone 1 element spacing is chosen to ensure that there are no grating lobes at the highest frequency of operation. The outer dimensions of each zone are successively doubled in progressing from Zones 1 to 3. Assuming that the AESA is required to operate over a 10:1 bandwidth, say from 1–10 GHz, then Zones 1 to 3 support grating lobe-free operation within frequency ranges of 1–10 GHz, 1–5 GHz and 1–2.5 GHz respectively. This implies that RF functions operating in the 1–2.5 GHz band may use the full array, namely, Zones 1 to 3; those functions operating at frequencies between 2.5–5 GHz are restricted to use of elements in Zones 1 and 2; and RF functions active in the 5–10 GHz band may only employ the Zone 1 elements. The total element count for the AESA in this case is about six times less than that which would be required if the array was fully populated with elements at spacing d = 0.54λ_g_. In addition to lower costs, this reduced element count also results in less AESA weight. However, the disadvantage of the wavelength-scaled array approach is that it reduces flexibility in configuring the AESA of a MFRF system. For example, the decreasing size of the array area available to RF functions as their operational frequency band increases leads to formed beams that have approximately the same minimum widths for all functions. This outcome may not have a significant impact though, as Table 1 indicates that beamwidth requirements for all RF functions are comparable. Another resulting restriction in AESA use occurs with formation of transmit beams. Because RF functions operating in higher frequency bands of the supported bandwidth are constrained to use a smaller area of the AESA, the ability to generate multiple simultaneous transmit beams from different parts of the array to accommodate these RF functions may be adversely affected.

Most of the analog components in the Tx and Rx channels have bandwidth limitations which may be additional drivers in a decision to use several AESAs to cover multiple frequency bands within the 0.5–40 GHz range of interest. These limitations are described as follows:

- Circulator: For a combined Tx/Rx array that requires use of a circulator, currently available microstrip circulators typically are designed to have bandwidths of 0.5–4 GHz and isolation values of 20–30 dB. There are a few wider-bandwidth circulators available, such as the UIY Model UIYBMC1212A (Shenzhen, China) [33]. This circulator has a bandwidth of 10 GHz, extending from 8–18 GHz, but only provides about 13 dB isolation between ports, which may be inadequate. Connectorized circulators can have bandwidths of up to 12 GHz [34], but these circulators are likely too bulky to be included in T/R modules, and have isolation values of only about 15 dB as well.
- Limiter: There are limiters available that cover the full frequency range of interest. An example is the 1GC1–8053 (Keysight Technologies, Santa Rosa, CA, USA) which is a MMIC diode limiter that covers 0–65 GHz, with power limiting beginning at 10 mW [35].
- LNA: A number of wideband GaAs MMIC LNAs are commercially available with good performance specifications, although there appears to be none that cover the full operational frequency range. For instance, the Analog Devices HMC1049LP5E maintains a gain of 15 dB with a noise figure of less than 4 dB over 0.3–20 GHz [25], while the Analog Devices HMC-ALH445 operates over 18–40 GHz with 9 dB gain and acceptable noise figure of less than 5 dB [36].
- HPA: The Table 2 lists some key specifications for several commercially available MMIC HPAs with different bandwidths and operating frequencies [37,38]. The first HPA listed uses GaAs technology, while all of the others in the table are GaN devices. As mentioned earlier, GaAs HPAs cannot produce power outputs as high as similar-sized GaN-based HPAs, but this one is included because a GaN device with a similar ultrawide bandwidth could not be found. The quantity PAE indicated in the table is *p*ower-*a*dded *e*fficiency, calculated as $\mathrm{PAE}=\left(P_{RF\_\mathrm{out}}-P_{RF\_\mathrm{in}}\right)/P_{DC\_\mathrm{in}}$, where $P_{RF\_\mathrm{out}}$ is the maximum HPA RF power output, $P_{RF\_\mathrm{in}}$ is the maximum RF power input, and $P_{DC\_\mathrm{in}}$ is the DC supply power required by the HPA. PAE represents the percentage of the DC supply power that is converted in the HPA to useful RF output power, with the remainder being dissipated as heat. All else being equal, a low value of PAE for the HPAs implies that a larger DC power supply must be provided for the AESA, and that the thermal cooling design for the array becomes more challenging. The table indicates that both HPA output power and PAE generally decrease as bandwidth and operating frequency increase. While the first HPA in the table covers the full frequency range of interest, its output power of 0.25 W and PAE of 10% are by far the lowest of the HPAs listed. As mentioned in Section 3.2.1, an output power of at least 10 W per element is generally required to accommodate high EIRP RF functions like naval radar, so this HPA would be unsuitable. The other HPAs listed may be appropriate MFRF system candidates, although they cover smaller bandwidths. The TGA2813 and TGM2635-CP (Qorvo, Greensboro, NC, USA) are designed specifically for S-band and X-band radars. Their favourable output power and PAE specifications highlight the benefits of potentially using narrowband HPAs in conjunction with a multiband AESA such as the dual-band array described above.

Regarding the digital receiver in the Rx channel of Figure 2, a critical specification is the number of bits of digitization that must be provided by the ADC to meet the highest dynamic range specification of 90 dB in Table 1. While ADC technology is evolving rapidly, ADCs with the roughly 15 bits required are not yet available at the 40 GSPS sampling rates needed to perform direct RF sampling over the full 0.5–40 GHz range of RF function operating frequencies. The current state-of-the-art in commercially available ADCs is represented by the entries in the table below [39]. It is observed that number of digitization bits decreases with increasing sampling rate. The 24-bit digitizer listed is applicable only for narrowband RF functions like naval radar. The ADC models with 14 and 16 bits may be suitable for all RF functions, while the 12-bit device could accommodate the ES function with its lower dynamic range specification of 60 dB. However, none of these ADCs have the bandwidth to allow direct RF sampling, except in the lower end of the operating frequency range. Consequently, an analog tuner would generally need to be included in the digital receiver in front of the ADC to translate the RF signal frequency down to an intermediate frequency that falls within the bandwidth of the ADC. This implies the need to have several AESAs or subarrays within the AESAs that are assigned to different bands which collectively cover the full operating frequency range, assuming that instantaneous coverage of the operating spectrum is an important goal (which would certainly be the case for the ES function).

Finally, the DWG in the Tx channel must be able to generate waveforms with instantaneous bandwidths as high as 1 GHz, based on the EA function requirements listed in Table 1. This capability appears currently achievable with commercially available technology. For example, the Analog Devices AD9914 includes both a DDS and a 12-bit DAC on the same board, and supports output waveform bandwidths up to 1.4 GHz [40]. From a performance perspective then, currently available DWG technology appears to pose no significant limitation with regard to use in a naval MFRF system.

As a general concluding observation, the discussion in this section points to ADC technology as representing the most severe impediment to wideband MFRF system implementation, given that the most stringent RF function dynamic range requirements can only be met when ADC bandwidths are restricted to 1–3 GHz.

#### 3.2.3. Element-Level vs. Subarray-Level Digitization/Waveform Generation

Section 3.1 discusses the key advantages of element-level digitization and waveform generation. The main deterrents to performing these operations at the element level for an AESA are added cost and design complexity, given that there are typically several thousand elements on an array and each element, if dual-polarized, would require two DDSs and two ADCs. As a rough order-of magnitude indication of incremental per-element costs, the Analog Devices DDS is listed at about $140 USD per unit, while the Texas Instruments ADCs in Table 3 (TI, Dallas, TX, USA) are priced in order of decreasing bandwidth as $2000 USD, $850 USD, $400 USD and $20 per ADC. There is an obvious correlation of decreasing price with narrower ADC bandwidths.

**Figure 6:**
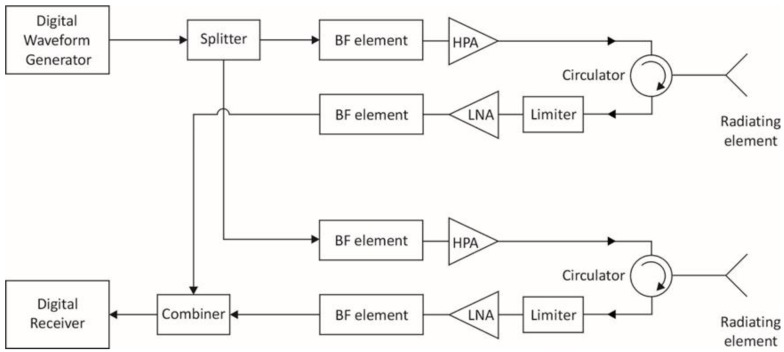
Subarray-level digitization and waveform generation.

The alternative approach to an element-level design is to divide the AESA into fixed subarrays, with each subarray, rather than each element, serviced by a single digital receiver and/or DWG. This is illustrated in Figure 6 for the simple case of a two-element subarray on a combined Rx/Tx AESA. In a typical implementation, the waveform signal from the DWG is split and injected into the Tx channels of the subarray, while the received signals in the Rx subarray channels are summed in a combiner before digitization by the digital receiver. Note that subarray sizes for Tx and Rx operations can generally be different, even with combined Tx/Rx arrays. Also, either digitization or waveform generation may be implemented at the element level, while the other is realized at the subarray level. For a subarray-level approach, analog *b*eam*f*orming (BF) elements must be included in the Rx and Tx paths. These may be phase shifters in the case of narrowband waveforms or more complex TTD circuits for wideband signals. A waveform can be considered narrowband from a subarray viewpoint if $L_{s}\ll c_{0}/2B$, where $L_{s}$ is the maximum dimension of the subarray, $c_{0}$ is the speed of light, and B is the waveform bandwidth [28]. For the waveform bandwidth of B=1 GHz that must be accommodated by the EA and ES functions, the subarray dimensions must therefore be much less than 15 cm to meet this narrowband criterion. However, these subarray dimensions would be comparable to the actual element spacing, given the frequency ranges of operation in Table 1, so larger subarrays with TTD beamforming elements must be used. TTD formation of Tx beams with maximum array gain is then accomplished by employing the DWGs to introduce relative waveform time delays between subarrays, and the TTD beamforming elements to generate additional relative waveform time delays between radiating elements within each subarray. For TTD formation of Rx beams, the TTD beamforming elements impose relative time delays between signals received from different elements within each subarray, and after combining and digitization at the subarray level, relative time offsets between the subarrays are added in the digital domain before coherent summation.

Compared to an element-level implementation, subarray-level digitization/waveform generation results in less flexibility to dynamically configure the AESA. Since the subarrays assigned to DWGs and/or digital receivers in a subarray-level implementation are essentially fixed in size by the hardware design, the RAM is restricted to partitioning the AESA into areas that are multiples of this smallest subarray size. If these subarrays are relatively large, this constraint may have an adverse effect on the ability of the AESA to accommodate multiple RF functions.

In the case of subarray-level digitization, a potentially more serious impact on MFRF system performance results from the loss of ability to digitally form multiple simultaneous Rx beams in different directions using signals received from the same set of AESA elements. With digitization at the subarray-level, only one Rx beam can be formed with the elements in a subarray, restricting the maximum possible number of simultaneous independent Rx beams to the total number of designated subarrays on the AESA. This may be problematic for some RF functions like ES and radar that benefit from the use of simultaneous Rx beams to provide rapid, if not instantaneous, coverage of a large surveillance area. Consequently, modern stand-alone naval radar systems are increasingly employing element-level digitization, especially since the cost/benefit trade-off in the case of these narrowband systems has become much more favourable due to the current availability of suitable low-cost ADCs, such as the Texas Instruments ADS1675 at a $20 USD unit price.

An alternate approach to subarray-level digitization/waveform generation is a fully connected architecture with hybrid beamforming [41]. This architecture reduces the number of digital receivers and/or DWGs but has greater flexibility than the use of fixed-size subarrays. For example, the use of Butler matrix-based analog beamforming on transmit could allow the beams to be changed dynamically depending on the channel condition and the number of dominant beams.

## 4. MFRF System Prototype Development Programs

MFRF systems have been slow to find their way into operational use, likely due to the technical challenges discussed in this paper, as well as perceptions of higher programmatic risk associated with procurement and deployment of such systems in comparison to multiple traditional single-purpose RF systems. However, the interest in MFRF systems remains high, and in particular, there have been three notable MFRF system prototype development programs conducted in recent years–the *A*dvanced *M*ultifunction *RF C*oncept (AMRFC) program, the *In*tegrated *Top*side (InTop) program and the *M*ultifunction *A*ctive *E*lectronically *S*teered *A*rray (M-AESA) program. These are discussed below.

### 4.1. AMFRC

#### 4.1.1. Overview

The AMRFC program was carried out from 1998–2009 by the US Naval Research Laboratory (NRL) under the sponsorship of the Office of Naval Research (ONR) [1,42]. Its goal was to demonstrate for the first time the concept of a MFRF system, with real-time radar, EW, EA and communications functions sharing usage of waveform generators, receivers and a single pair of separated Rx and Tx AESAs. The main contractors involved were Raytheon [43] (Waltham, MA, USA) and Northrop Grumman (Falls Church, VA, USA). Lockheed Martin (Bethesda, MD, USA) was responsible for the Rx array and digital receivers, Raytheon built the real-time signal/data processor, operator display system and the portion of the DWGs that produced the digital waveform samples, and Northrop Grumman provided the Tx array and the DACs for the DWGs.

After development and integration, the AMRFC testbed was installed on a cliff top at Chesapeake Bay (MD, USA). Throughout 2004, trials were conducted to demonstrate the unique capability of the system to simultaneously maintain radar surveillance of the area, intercept threat emissions using its ES function, jam threat radars with the appropriate EA technique, and establish and maintain SATCOM and terrestrial CDL communication links. Surface vessels provided targets of opportunity for the radar function, while RF simulators located on Tilghman Island in Chesapeake Bay and aboard the NRL P3 test aircraft emulated threat radars and active missile seekers to exercise the ES and EA functions. CDL terminals on the island and aboard the test aircraft supplied the means to establish terrestrial communication links with AMRFC. 

ONR had hoped that the AMRFC technology would be transitioned to the US Navy’s new DDG 1000 destroyer that was about to begin development in 2005. However, despite the technical success of the AMRFC program, the overall MFRF system technology was deemed to still be too immature to move directly into an acquisition program. This was reflected in the assessment that the AMRFC testbed was at best at *T*echnology *R*eadiness *L*evel (TRL) 6, whereas TRL 7 is considered to be the minimum level required for a new technology to be considered ready for operational deployment. Another issue was that the US defence funding and acquisition process has traditionally been “stove-piped” into separate radar, EW and communications areas, which was not conducive to acquisition of multifunction systems. The only component of the AMRFC program that was immediately adopted for operational use was some of the technology associated with the ES function, which was further refined to TRL 7 as the Multifunction EW (MFEW) Advanced Development Model (ADM). From 2005 to its conclusion in 2009, the AMRFC program carried on with reduced resources, focusing mainly on continued development of enabling technologies in the area of digital arrays and RF components, particularly HPAs.

The total cost of the AMRFC program was in excess of $200 M USD, including the cost of the MFEW ADM development, and at its peak, involved more than 200 people, including both government personnel and industry contractors.

#### 4.1.2. Technical Description

The system was designed to operate over 6–18 GHz. The original AMRFC design was based on a lower band, with an emphasis on the radar function. However, the US Navy decided early in the development to prioritize demonstration of modern EW and communication capabilities, which was better accommodated by the high band design. The AMRFC testbed design represents the trade-offs of Section 3 that were made in this case, based on the state-of-the art in RF and digitizer technologies at that time [44]. Other design decisions were driven by the goal to demonstrate real-time operation, given the limitations in processing power in the late 1990s compared to today. Key features of the AMRFC testbed design are described below.

- The separate Rx and Tx arrays were each approximately 32 cm square, and populated with wideband dual-polarized radiating elements based on orthogonal flared notches. The element spacing was set on the Tx array to ensure grating lobe-free operation up to 18 GHz over a scan volume of ±50° in azimuth/elevation. The arrays had a centre-to-centre separation in the same plane of about 3.7 m to ensure sufficient EM isolation when transmitting and receiving simultaneously.
- The Tx array had 1024 elements, segmented into four quadrants of 256 elements each, with each quadrant further subdivided into four subarrays to yield a total of 16 subarrays. There was a RF Tx module behind each subarray, comprising a HPA and a pair of RF channels (one per polarization) feeding the subarray elements, with full amplitude and phase control provided in each polarization path. The HPA was a GaAs device, capable of generating several watts across the operating band in either linear or saturated modes. Note that with this relatively low HPA power, combined with the small Tx array size, the EIRP was only high enough to allow demonstration of a radar function equivalent to a short-range navigation radar, rather than the naval radar functions of Table 1; this was a reflection of program priorities, as well as limitations in cost and HPA technology at that point in time. There was a separate DWG allocated to each array quadrant, where each DWG included a DRFM component for coherent EA, and was capable of generating waveforms of up to 1 GHz bandwidth. By using photonic switches, each DWG could be routed to any or all of the four array quadrants for maximum flexibility. This configuration allowed the formation of Tx beams using any combination of quarter, half or full array, up to a maximum of four independent simultaneous beams (one per quadrant).
- The Rx array had 1152 elements in total, grouped into nine 128-element subarrays, with an Rx module behind each element. Each Rx module had four independent RF receive channels: three linearly polarized channels, which were each fed by one of the two orthogonal flared notches of the dual-polarized element, and one polarization agile channel, which carried the sum of the two polarization signals from each element. It appears that there were no beamforming elements in the Rx channels. The Rx channel data was utilized as follows.
  ⚬ The RF signals from nine elements arranged in an interferometer configuration on the Rx array were downconverted and routed to a remote ES processor for precision direction-finding of narrowband strong emitters. This involved processing the phases between the nine inputs using interferometric algorithms to compute azimuth and elevation. The interferometric approach, while only feasible for strong emitter signals, has the advantages of covering a wide range of operating frequencies and wide field-of-view. The RF signals from two other elements were provided directly to auxiliary receivers for potential use by the DWG DRFMs in support of coherent EA techniques.
  ⚬ The RF signals from all the Rx modules in each subarray were combined downstream on a channel basis, that is, all of the Channel 1 signals from the Rx modules in a subarray were combined, all of the Channel 2 signals were combined, etc. These combined signals were then provided on four ports on each of the nine subarrays for the following processing.
    ◾ The RF signals from three of the four subarray ports on each of the nine subarrays were sent to three nine-channel analog beamformers (i.e., one beamformer per port) for SATCOM links. Since beam pointing angles for SATCOM links change slowly, the capability afforded by digital beamforming to rapidly change beam direction was not needed, and the load on digital processing resources could consequently be reduced by the use of analog beamformers.
    ◾ The RF signals from three subarray ports on each subarray were provided to narrowband digital preprocessors, where they were downconverted to IF, sampled with 14 bits at 60 MHz and passed on to three nine-channel digital beamformers (i.e., one beamformer per port), where beams were computationally formed by phase shifting. Up to four simultaneous Rx beams per narrowband beamformer could be formed, resulting in a total of up to 12 beams. These beams were used for radar, CDL communication links, and ES.
    ◾ The RF signals from two subarray ports were provided to wideband digital preprocessors, where they were downconverted to IF with a bandwidth of 230 MHz, sampled with 8 bits at 960 MHz, digitally downconverted to a complex baseband signal, and passed on to two nine-channel digital beamformers implemented with vector processors. Up to two simultaneous beams per beamformer could be formed, where TTD processing was employed. These beams were mainly used for ES surveillance of weak emitter signals, where the detection of such signals benefits from the Rx array beamforming gain. As mentioned in Section 3.1, the wideband digitization and TTD beamforming accommodates detection of emitter signals with large instantaneous bandwidth.

As a point of interest, the design decision in the AMRFC program to implement only subarray-level Rx beamforming without element-level beamforming capability has the following implications: (1) With only nine inputs to each beamformer, the maximum coherent Rx array gain was only ≈19 dB; (2) The presence of grating lobes in the beamformed Rx array pattern was effectively determined by the centre-to-centre subarray spacing, rather than the element spacing. The subarray separation was about 11 cm, which was larger than 0.54λ for all frequencies within the operating band of 6–18 GHz. Consequently, grating lobes at scan-off angles were likely an issue. The lack of any element-level beamforming in the design is an unusual decision that may have been motivated by cost, and perhaps by a conclusion that this feature was unnecessary for the purposes of demonstrating the benefits of MFRF systems.

### 4.2. InTop

#### 4.2.1. Overview

The ONR-sponsored InTop program was initiated in 2009 as a follow-on to AMRFC and is still ongoing. It has the goal of further advancing wideband array and RF component technology for use in MFRF systems, based on modular, scalable, open RF architecture [42,45]. The main InTop effort involves the demonstration of such technology through the development of five RF system prototypes, each with less MFRF capability than that designed into the AMRFC testbed, but ideally with higher TRL. Given the obstacles to transitioning the more ambitious AMRFC into operation, this was seen to be a more prudent approach that would facilitate spinning off demonstrated core capabilities into acquisition programs, similar to the path followed by the MFEW project. The five prototypes are briefly summarized as follows. A more detailed technical description is provided in subsequent subsections.

- *MFEW ADM*: The MFEW ADM was largely developed under AMRFC, but was completed under the InTop program. Northrop Grumman was the industry lead on this development. As initially mentioned in Section 4.1.1, the MFEW ADM was based on some of the ES functionality incorporated in the AMRFC testbed. The MFEW technology has subsequently been transitioned to the US Navy’s *S*urface *EW I*mprovement *P*rogram (SEWIP) Block 2 acquisition program [46].
- *EW/IO/Comms ADM***:** This ADM supported EA, *i*nformation *o*perations (IO), and *l*ine-*o*f-*s*ight (LOS) terrestrial communications using a common set of AESAs and RF subsystems. Northrop Grumman was the prime contractor for this work, with announced contracts totalling $87 M USD [47]. The ADM has been completed, and the technology has transitioned to the US Navy’s SEWIP Block 3 acquisition program.
- *Submarine Wideband SATCOM Antenna Subsystem*: This subsystem involved a set of AESAs that was designed for mounting on a submarine mast to provide the capability for simultaneous SATCOM links in different bands. Lockheed Martin was awarded the development contract worth roughly $32 M USD [48], and has completed the work. The technology is being transferred to the *Adv*anced *H*igh-*D*ata-*R*ate (AdvHDR) submarine SATCOM acquisition project. The work is also applicable to SATCOM for ships.
- *LowRIDR ADM*: The *Low*-band *R*F *I*ntelligent *D*istribution *R*esource (LowRIDR) ADM aims to consolidate several RF functions that operate in a low frequency band, including communications, EA and ES, into a common set of antennas and related hardware. The ADM is not yet completed.
- *FlexDAR ADM*: The primary RF functions that are included in The *Flex*ible *D*istributed *A*rray *R*adar (FlexDAR) ADM are radar, EA and ES. A missile data link capability is also provided. Raytheon is developing the FlexDAR arrays under contract to ONR [48], including the associated Rx and Tx channels, while NRL is providing the back-end functionality, such as the RAM and signal/data processing. The FlexDAR concept actually comprises two systems that will be network-linked together to also demonstrate the benefits of multistatic radar operation in the form of improved detection, tracking and electronic protection. The ADM is scheduled for completion in the 2018–2019 time frame.

#### 4.2.2. MFEW ADM

The MFEW ADM antenna utilized 20 dual-polarization sinuous receive elements arranged in an interferometer configuration. Sinuous elements are planar with a circular shape [49]. They feature a low RCS, a bandwidth as high as 9:1, a large element beamwidth, and a phase centre that is stable with frequency, all of which are desirable for interferometer applications. (However, sinuous elements have a relatively large diameter, making them unsuitable for use in AESAs, where half-wavelength inter-element spacing is required to avoid grating lobes when beamforming. For example, the Randtron Antenna Systems (Menlo Park, CA, USA) Model 53640 sinuous antenna element [50] covers a wide frequency range of 2–18 GHz, but has a physical diameter of 6 cm. In an AESA, this would imply a minimum inter-element spacing of 6 cm, which would result in potential beamformer grating lobes for all frequencies above 2.7 GHz).

Each antenna element had an associated tuner and digital receiver which captured a signal bandwidth of 400 MHz. The digitized signals from each receiver were then filtered with a bank of 32 MHz digital filters before detection processing to maximize sensitivity while minimizing the effects of external interference. The tuners were employed in a scanning architecture to cover all frequency bands of interest. The scanning process utilized *a priori* information about emitter parameters and frequency concentration to optimize overall response time of the ES function.

Determination of AoA was done with 14 of the antenna elements which formed an L-shaped pattern. This arrangement essentially provided two orthogonal interferometers to allow computation of both azimuth and elevation of an emitter signal. A RAM dynamically allocated these antenna elements to either frequency scanning or AoA determination tasks as required.

As discussed in Section 2.2, implementation of the ES function without an AESA and the associated advantages of beamforming generally limited the system to detection of stronger emitter signals.

#### 4.2.3. EW/IO/Comms ADM

The EW/IO/Comms ADM utilized one array set per quadrant of surveillance volume, where each array set consisted of a Rx and Tx AESA [16]. Each Tx array provided up to four independent beams, while each Rx array supported from four to 16 independent beams through the use of four Rx channels per element, as was the case with the AMRFC testbed design.

The specifications of the Tx AESA and associated channel components were driven mainly by the EA function requirements, the most important being an operating frequency range from C band to K_a_ band, and sufficient EIRP to provide self-protection for a platform with a large RCS. The design also included an interface with the ship’s ES system, the information from which was used by the EA function to track hostile emitters in angle, and aid in design of the jamming techniques.

The IO functionality was provided through interfaces to the ship’s signal exploitation equipment that provides threat identification information. The EA function incorporated this information in its response. The communications requirements for the ADM included two independent legacy system X-band CDL links, at least four independent TCDL links, and a K_u_-band network communications waveform within each quadrant covered by an array set.

#### 4.2.4. Submarine Wideband SATCOM Antenna Subsystem

The submarine SATCOM antenna subsystem employed separate Tx and Rx arrays to supply SATCOM services from C-band through to V-band. It supported from four to at least eight simultaneous communication links.

#### 4.2.5. LowRIDR ADM

The frequency range of operation for the lowrider ADM is VHF to C-band. Communications, EA and ES functions are supported throughout this frequency range. In the case of the communications function, the focus is on line-of-sight terrestrial communications, specifically Link 16, Identification Friend or Foe (IFF) and Tactical Air Navigation (TACAN) [51].

#### 4.2.6. FlexDAR ADM

The two AESA-based FlexDAR ADM systems operate only in S-band, which is a typical band for the radar function. Consequently, while EA and ES functions are included in FlexDAR, their implementation is restricted to this relatively narrow band of frequencies. The FlexDAR design features element-level digitization of the AESA Rx channels.

### 4.3. M-AESA

#### 4.3.1. Overview

The M-AESA program was a joint Sweden-Italy initiative aimed at developing new technology and system concepts for a next generation AESA-based MFRF system that integrated radar, EA, ES and communication functions [52]. The ultimate goal was to be able to potentially insert this technology into future Swedish and Italian ground, air and naval platforms, utilizing common RF hardware modules. The industrial consortium of Saab Microwave Systems AB (Stockholm, Sweden), Selex Sistemi Integrati (Rome, Italy) and Elettronica Group (Rome, Italy) was awarded the contract in 2005 to conduct this program.

There were three program phases:

- *Phase 1* (2005–2006): Technology concept/application formulation–analysis of existing system and related technology base to outline potential future system applications.
  *Deliverables:* (1) Statement of Work (SOW) and Work Breakdown Structure (WBS) for Phase 2, and (2) schedule and cost estimates for Phases 2 and 3.
- *Phase 2* (2006–2010): Concept refinement–development of RF building blocks, selection of architecture for the M-AESA system.
  *Deliverables:* M-AESA system prototype at TRL 4.
- *Phase 3* (2011–2014): Technology development.
  *Deliverables:* M-AESA system prototype at TRL 6.

Work completed in Phases 1 and 2 was reported in the open literature and is summarized in the next section. 

#### 4.3.2. Technical Description

The M-AESA system provided Tx functionality from 4.5–18 GHz and Rx functionality from 2–18 GHz. The extended frequency coverage at the low end of the spectrum for Rx, as compared to Tx, was to accommodate certain communication services.

Two main antenna configurations were considered for the M-AESA program for the notional ship-mounted case [52]. The first configuration involves a wideband combined Rx/Tx AESA for each quadrant that is shared by radar, EA, ES and communication functions. It is representative of the ideal MFRF system architecture presented in Section 3.1, with all of its potential benefits. The second antenna layout comprises: a multiband or at least more narrowband combined Rx/Tx array that is utilized by radar functions and some in-band communication services; a smaller wideband Tx array for use by the EA function and the Tx portion of communication links; and a linear wideband Rx array to support ES and the Rx side of communication links. Note that beams formed with the linear array are narrow in azimuth but wide in elevation, so that AoA determination in the ES function is restricted to the azimuth dimension. Referring to the trade-off discussions in Section 3.2, the second configuration ensures sufficient isolation between Rx and Tx channels during periods of simultaneous reception and transmission. It also allows components of narrower bandwidth to be used at least for the radar function, with attendant advantages such as the availability of higher power HPAs and faster ADCs with higher dynamic range. It appears that this second configuration was ultimately selected, based on the developed RF components described below.

The primary RF building blocks developed under the M-AESA program were wideband antenna arrays [28] and wideband T/R modules incorporating analog TTD beamforming elements [53,54]. The wideband arrays were based on the type of flared notch element depicted in Figure 3. A test array was fabricated during Phase 2 of the M-AESA program [28]. It consisted of 25 × 25 dual-polarized elements spaced about 1.5 cm apart, which provided grating lobe-free beamforming up to 10.5 GHz for scan angles within ±60°. The reflection coefficient for the centre element of the array was measured to be less than −10 dB over 2–18 GHz and over all scan angles.

**Figure 7:**
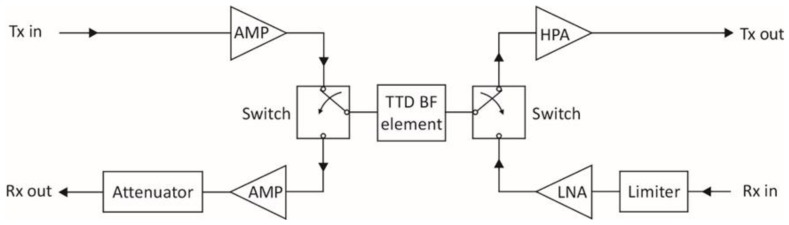
Simplified block diagram of M-AESA T/R module.

The simplified block diagram of the T/R module is presented in Figure 7. The wideband *amp*lifiers (AMP), switches, attenuator, and TTD beamforming element were packaged together as a GaAs MMIC core chip. The entire T/R module had dimensions of 1.4 cm wide × 5 cm long × 0.4 cm thick. The 1.4 cm width requirement was a challenging one that was set by the specification for 1.5 cm element spacing in the array.

The switches served to select between Rx and Tx paths, with isolation of greater than 40 dB provided over 2–18 GHz. Note that this design precluded the possibility of simultaneous reception and transmission within the same T/R module. The depicted arrangement of the two switches allowed the same TTD beamforming element to be used by either Rx or Tx signals. The TTD beamforming circuit provided up to 124 ps of time delay (equivalent to 3.7 cm in free space) by switching between “artificial” transmission lines realized with inductor-capacitor networks. This approach was used because physical transmission lines would have occupied an unacceptable amount of chip area for the required delay. The delay was controlled with a 5-bit word, where the least significant bit was equivalent to 4 ps of delay. The attenuator in the Rx chain was included to allow for tapering of the Rx signal across the array, which might have been desired for sidelobe suppression during beamforming. The purpose of the wideband amplifiers in both the Rx and Tx channels (to the left of the TTD element in Figure 7) was to compensate for insertion losses introduced by the switches, attenuator and, mainly, the TTD beamforming element. The HPAs were GaAs MMIC devices, with their characteristics indicated in Table 4 for two different models that were developed. HPA 1 was aimed at use in transmit modules for the wideband arrays in the second antenna configuration, while HPA 2 was intended for T/R modules that would be employed for the radar array in that configuration. While the operating frequency range of HPA 2 was somewhat less than that of HPA 1, it was still large enough to accommodate a C-band or X-band radar function. The obvious advantage of using HPA 2 for radar functions was the higher output power. These HPA specifications compared favourably to those of similar devices on the market in the 2010 time frame that the M-AESA HPAs were developed. However, the emergence of GaN technology since that time has resulted in currently available HPAs that are significantly superior, as indicated by comparisons to the HPA specifications in Table 2. Lastly, the wideband LNA used in the T/R module design yielded an overall measured noise figure for the module of less than 4.1 dB over the entire Rx operating frequency range of 2–18 GHz, and less than 2.9 dB over typical radar operating frequencies of 6–13.5 GHz.

The M-AESA design utilized subarray-level waveform generation and digitization. Consequently, the total time delays required for TTD beamforming were achieved by a combination of digital domain implementation at the subarray level, and analog delays at the element level. Specifically, the design called for the capability to provide analog delays of up to 1144 ps (equivalent to 34 cm in free space) for the signals from/to each array element. As mentioned previously, a maximum of 124 ps of delay was available in each T/R module, where this number was likely constrained by the available space on the core chip for the delay line implementation. Thus, to meet the analog delay requirement, a separate analog TTD board that serviced each subarray provided up to an additional 1020 ps of delay for each array element, with the delay controlled by an 8 bit word with the least significant bit equivalent to 4 ps. The TTD board contained the same core chip as in the T/R module to provide the first 5 bits of delay, and then microstrip transmission lines were employed to implement the higher order 3 bits. Switches were also included on the board to select between Rx and Tx modes.

## 5. MFRF System Resource Management

As discussed in the previous sections, the design trade-offs that prevent realization of the ideal MFRF system architecture are driven primarily by existing performance limitations and/or costs of hardware technologies related to array radiating elements, ADCs, and RF components such as HPAs, LNAs and circulators. Consequently, the continued advancement of these technologies will be the most significant factor in enabling the optimal design and cost-effective deployment of naval MFRF systems. It will be valuable to maintain a technology watch in these areas with the aim of identifying future MFRF system design concepts that can exploit advances in the underlying hardware technology. The technology watch effort would involve monitoring the available literature to recognize relevant emerging technology trends, and periodically surveying commercial-off-the-shelf products to establish current state-of-the-art.

A key area of future research in MFRF systems is resource management, a task which is executed by the Resource Allocation Manager (RAM) depicted in the block diagram of Figure 1. In the past, most research in RAM architectures and algorithms as they pertain to RF systems has focused on resource management for phased array radars, since radar comprises a number of functions, and historically was the first and largest application of phased arrays. There has been significant previous work carried out in adaptive *r*adar *r*esource *m*anagement (RRM) [55,56,57]. This work provides a solid foundation for investigating RAM implementations for MFRF systems, since many of the techniques studied for RRM are somewhat generic in their application. The challenge in extending this work to MFRF systems lies in the fact that not only must more RF functions be accommodated within the set of shared electronics and AESAs, but some of the additional functions also have priorities as high as or even higher than those of the radar functions. For example, the ES function must always be allocated a portion of system resources to enable continuous monitoring for threat emissions, and the EA function, when active, commands the highest priority due to its critical self-protection role. Consequently, a careful study of resource management techniques for MFRF systems is required to determine the extent to which potentially suboptimal resource allocation to any single RF function during system overload conditions may impact their performance.

Signal processing for RF functions is another important area of future research. This topic is not discussed here due to space constraints. Instead, the reader may consult references on signal processing for radar [58], ES [59], EA [60], and communications [61]. Based on the above observations, key elements of resource management for MFRF systems are presented below.

### 5.1. Development of Modelling and Simulation Capability

Evaluation of resource management techniques for multifunction systems in complex scenarios relies heavily on modelling and simulation. A modelling tool for resource management would need to include the following capabilities:

- model for a real-time scheduler that accounts for radar, ES, EA and communications tasks.
- modelling of radar search and track modes.
- dynamic arbitrary partitioning of arrays to accommodate operation of multiple RF functions that in general are activated at different points in time.
- separation of Rx and Tx modes to provide the flexibility of utilizing different transmit/receive array gains and beamwidths, as would be the case if a MFRF system was configured with separate Rx and Tx arrays for example.
- inclusion of threat emitters in the scenario to stimulate the ES function.
- modelling of ES, EA and communication functions, including hand-off of threat emitter information from the ES to the EA function.

With regard to the last item, it would likely suffice to implement relatively simple models for ES, EA and communications functions, since the purpose of the study would be to evaluate resource management techniques rather than the effectiveness of specific waveforms or algorithms used in these functions. For example, when EA is activated in the model, it can simply be assumed that suitable jamming waveforms are being used without the need to explicitly model them. The focus instead would be on modelling the action taken by the RAM at that instant to provide the EA function with sufficient system resources to transmit at the required EIRP for the length of time deemed necessary to likely defeat the threat. In the case of ES, modelling of the emission detection process would be implemented in a similar fashion to the radar range equation-based scheme utilized for modelling radar detections, except that a one-way version of the radar range equation would be used in the case of ES. Beyond that, modelling of the ES function would include assignment of system resources by the RAM to allow continuous monitoring for threat emissions across the frequency spectrum, and timely coverage of the required surveillance volume with a receive beamwidth narrow enough to achieve required AoA measurement accuracies. 

### 5.2. Development and Evaluation of Resource Management Techniques

Once a suitable modelling and simulation tool has been developed, it would be used to explore the capabilities and limitations of different RAM schemes for a MFRF system. Resource management techniques that have been found to be effective for RRM would be good candidates for initial investigation. If necessary, these RRM techniques may be modified to optimize them for the MFRF system application. Also, new approaches may additionally be developed and tested.

The specific configuration and performance characteristics of the MFRF system hardware assets that are under RAM control are key factors in ultimately determining the extent to which resource management techniques can mitigate RF function performance degradation in challenging threat scenarios. Consequently, careful effort would be required in selecting a MFRF system architecture for this study which will be representative of the technology available within the timeline of interest. Based on the discussions in Section 3, a preliminary recommendation is that the modelled baseline MFRF system architecture include the following high-level features:

- *Separated Rx and Tx arrays*: For the foreseeable future, this is the only configuration that ensures sufficient isolation between Rx and Tx channels to allow simultaneous transmission and reception at the same frequencies, which is a requirement when ES and EA functions are involved.
- *Multiband*: Given the bandwidth limitations of a number of the required hardware components, it is prudent to assume a multiband system that covers the full operating frequency range up to 40 GHz with several bands. Examination of currently available technology described in Section 3 suggests that the performance characteristics of key components, namely HPAs, LNAs and ADCs, are good enough now or will be in the near future to support one such band that roughly covers from 4–10 GHz. This frequency range is significant, because it notionally encompasses all of the radar subfunctions (VS, HS and TI), as well as the X-band communication function, and an important part of the operating range for EA and ES functions. Thus, this band provides the greatest potential to yield a challenging overload situation for a RAM, and would consequently be the focus of the resource management study.
- *Element-level digitization and subarray-level waveform generation*: The flexibility to arbitrarily partition Rx arrays and digitally form any given number of multiple simultaneous Rx beams from the same part of the AESA are key motivations to using element-level digitization in a MFRF system. Element-level digitization has already been implemented in some commercially available radars, and the continuing strong trend in ADC technology towards higher performance and lower cost suggests that this design feature will be increasingly utilized in the near future. On the other hand, the benefits of element-level waveform generation are not as significant. Utilization of subarray-level waveform generation sacrifices some flexibility in configuring the Tx array, as discussed in Section 3, and requires the insertion of passive phase shifters or TTD circuits in the Tx channels to perform beamforming, but these disadvantages are likely outweighed by the reduction in system cost and complexity achieved by implementing fewer DWGs.

The modelling effort would include at least two scenarios–benign and challenging. These are described as follows.

- *Benign scenario*: In this baseline scenario, there would be no threats to which the MFRF system would need to respond. Consequently, the only functions activated would be ES, VS/HS radar, and occasionally X-band communications. The RAM would be able to allocate sufficient resources to each of these functions to allow them to operate at full performance.
- *Challenging scenario*: A number of threats would be inserted in this scenario to force the activation of EA and TI radar functions in addition to the functions operating in the benign scenario. Under these overload conditions, it is expected that the ideal amount of resources required by each RF function would not be available at every point in the scenario. The ability of the RAM to optimally allocate resources in this situation would be a key determinant of RF function performance.

## 6. Conclusions

This paper discusses the key issues that must be considered in the design and development of an AESA-based MFRF system that replaces a number of single-purpose RF systems and associated topside antennas on a modern naval vessel. The RF functions that are candidates for consolidation within a MFRF system are radar, EA, ES and communications. Radar subfunctions comprise Volume Search, Horizon Search, and Terminal Illumination. Communication services that are suitable for inclusion in an AESA-based MFRF system are X-band, K_u_-band and K_a_-band SATCOM, as well as K_u_-band TCDL.

The key transmit and receive requirements of the candidate RF functions were reviewed, namely, frequencies of operation, signal bandwidth, dynamic range, EIRP, one-way beamwidth, duty cycle, and signal polarization. These particular requirements pose the greatest challenges to development of a system that allows sharing of AESA radiating elements and RF components between multiple RF functions. Factors that drive these requirements for each RF function were also discussed.

An ideal MFRF system design architecture was presented that would accommodate the requirements of the individual RF functions, minimize the number of required AESAs and provide the maximum flexibility to facilitate dynamic assignment of system resources to these functions by a resource allocation manager. The key features of this ideal architecture are: (1) each radiating element in an AESA is shared by a transmit and receive channel, where generally each of the two channels can be used by a different RF function at the same time; (2) the AESA radiating elements are dual orthogonally polarized to allow reception/transmission at all signal polarizations; (3) all components have sufficient bandwidth to support the full operating range of frequencies for the RF functions of interest; and (4) each transmit channel and receive channel has its own digital waveform generator and digital receiver respectively to fully capture the benefits of element-level digitization and waveform generation. Currently, costs and/or performance limitations in existing hardware technology result in design trade-offs and compromises that prevent achievement of this ideal architecture. The trade-offs fall into three categories (1) combined vs. separate transmit/receive arrays; (2) wideband vs. multiband operation; and (3) element-level vs. subarray-level digitization/waveform generation. The considerations involved in these trade-offs were discussed in detail. The consequences of departure from the ideal MFRF system architecture are generally a larger number of required AESAs, and less flexibility in assigning AESA resources to the different RF functions.

A description was provided of MFRF system prototype development programs that have been conducted in other countries. The AMRFC program, which was carried out from 1998–2009 under the sponsorship of the US ONR, demonstrated for the first time the concept of a MFRF system covering 6–18 GHz, with real-time radar, EW, EA and communications functions sharing usage of waveform generators, receivers and a single pair of separated receive and transmit AESAs. The InTop program was initiated by ONR in 2009 as a follow-on to AMRFC and is still ongoing. It has the goal of further advancing wideband array and RF component technology for use in MFRF systems through the development of five RF system prototypes, each with less MFRF capability than the AMRFC testbed, but with higher TRL to facilitate spin-off into acquisition programs. The M-AESA program was a joint Sweden-Italy effort conducted from 2005–2014. Its aim was to develop and implement new hardware technology and system concepts in a next generation AESA-based MFRF system prototype that integrated radar, EA, ES and communication functions over a 2–18 GHz operating range. The primary RF building blocks that emerged from the M-AESA program were wideband antenna arrays and wideband T/R modules incorporating analog TTD beamforming elements.

Finally, MFRF system resource management was presented as an important future area of research. A specific emphasis of this work would be the effectiveness of resource management techniques to mitigate the impact of a system overload condition, where all RF functions must be activated in response to the detected threat scenario. Under these circumstances, it is assumed that there may not be sufficient system resources to ensure that the optimal amount of resources required by each RF function would be available at every point in the scenario. Evaluation of proposed resource management techniques in both benign and overload scenarios would be accomplished largely through modelling. The critical capabilities of a modelling and simulation tool for MFRF resource management were identified. A MFRF system architecture was proposed for use in the modelling effort, largely based on hardware technology that is available now or will likely be so in the near future. The key features of this configuration are separated multiband transmit/receive arrays, with element-level digitization and subarray-level waveform generation.

## Figures and Tables

**Table 1:** Comparison of transmit/receive requirements for naval RF functions.

RF Function	Frequencies of Operation (GHz) *	Signal Bandwidth (MHz)	Dynamic Range (dB)	EIRP (dBW)	One-Way Beamwidth (deg)	Duty Cycle (%)	Signal Polarization
Radar–volume search	L-band or S-band	2	90	S-band: 90 L-band: 75	2	20	Linear (V)
Radar–horizon search	S-band or X-band	5	90	90	2	20	Linear (V)
Radar–target illumination	X-band	negligible	N/A	90	N/A	≤100	Linear (V)
Electronic support	0.5–40	1000	60	N/A	1	N/A	All
Electronic attack	0.5–40	1000	N/A	50	N/A	≤100	All
Comms–X-band SATCOM	7.3–7.8 (Rx) 7.9–8.4 (Tx)	125	70	55	2	≤100	Circular (Tx/Rx orthogonal)
Comms–K_u_-band SATCOM	10.7–12.8 (Rx) 13.8–14.5 (Tx)	55	70	65	1	≤100	Linear (Tx/Rx orthogonal)
Comms–K_a_-band SATCOM	19.2–21.2 (Rx) 29.0–31.0 (Tx)	125	70	65	0.5	≤100	Circular (Tx/Rx orthogonal)
Comms–TCDL	14.4–14.8 (Rx) 15.2–15.4 (Tx)	300 (Rx) 90 (Tx)	70	45	2	≤100	Circular

* See more details on frequency band designations in Table A1.

**Table 2:** Representative specifications for HPAs [37,38].

Device	Frequency (GHz)	Gain (dB)	Output Power (W)	PAE (%)
Analog Devices HMC930A	0–40	13	0.25	10
Analog Devices HMC1087F10	2–20	11	7	20
Analog Devices HMC8205	0.3–6	26	40	38
Qorvo TGA2590	6–12	35	30	25
Qorvo TGA2813	3.1–3.6	22	100	55
Qorvo TGM2635-CP	8–11	26	100	35
Qorvo TGA2595	27.5–31	23	9	24

**Table 3:** Representative specifications for ADCs.

Device	Number of Bits	Sampling Rate (GSPS)
Texas Instruments ADC12DJ3200	12	6.4
Texas Instruments ADC32RF45	14	3.0
Texas Instruments ADS54J60	16	1.0
Texas Instruments ADS1675	24	0.004

**Table 4:** Specifications for M-AESA HPAs.

Device	Frequency (GHz)	Gain (dB)	Output Power (W)	PAE (%)
HPA 1	4.5–18	19	2 (CW and pulsed)	25–30%
HPA 2	5–12	20	4 (pulsed)	25–30%

## Appendix A

**Table A1:** IEEE frequency band designations.

Band	Frequency Range
HF	3–30 MHz
VHF	30–300 MHz
UHF	300–1000 MHz
L	1–2 GHz
S	2–4 GHz
C	4–8 GHz
X	8–12 GHz
K_u_	12–18 GHz
K	18–27 GHz
K_a_	27–40 GHz
V	40–75 GHz
W	75–110 GHz
mm	110–300 GHz
